# The Hallmarks of Ageing in Microglia

**DOI:** 10.1007/s10571-025-01564-y

**Published:** 2025-05-19

**Authors:** Laura Carr, Sanam Mustafa, Lyndsey E. Collins-Praino

**Affiliations:** 1 School of Biomedicine, The University of Adelaide, Adelaide, Australia; 2 Australian Research Council Centre of Excellence for Nanoscale Biophotonics, The University of Adelaide, SG31, Helen Mayo South, Adelaide, SA 5005 Australia

**Keywords:** Microglia, Ageing, Morphology, Neuroinflammation, Inflammageing, Immunosenescence

## Abstract

As ageing is linked to the development of neurodegenerative diseases (NDs), such as Alzheimer’s Disease and Parkinson’s Disease, it is important to disentangle the independent effect of age-related changes from those due to disease processes. To do so, changes to central nervous system (CNS) cells as a function of advanced age need better characterisation. Microglia are of particular interest due to their proposed links with the development and progression of NDs through control of the CNS immune response. Therefore, understanding the extent to which microglial dysfunction is related to phyisological ageing, rather than a disease process, is critical. As microglia age, they are believed to take on a pro-inflammatory phenotype with a distinct dystrophic morphology. Nevertheless, while established hallmarks of ageing have been investigated across a range of other cell types, such as macrophages, a detailed consideration of functional changes that occur in aged microglia remains elusive. Here, we describe the dynamic phenotypes of microglia and evaluate the current state of understanding of microglial ageing, focusing on the recently updated twelve hallmarks of ageing. Understanding how these hallmarks present in microglia represents a step towards better characterisation of microglial ageing, which is essential in the development of more representative models of NDs.

**Graphical Abstract:**

Microglial changes across different stages of life. Microglia have diverse functions throughout life; however, the characterisation of ageing hallmarks in microglia has been inconsistent

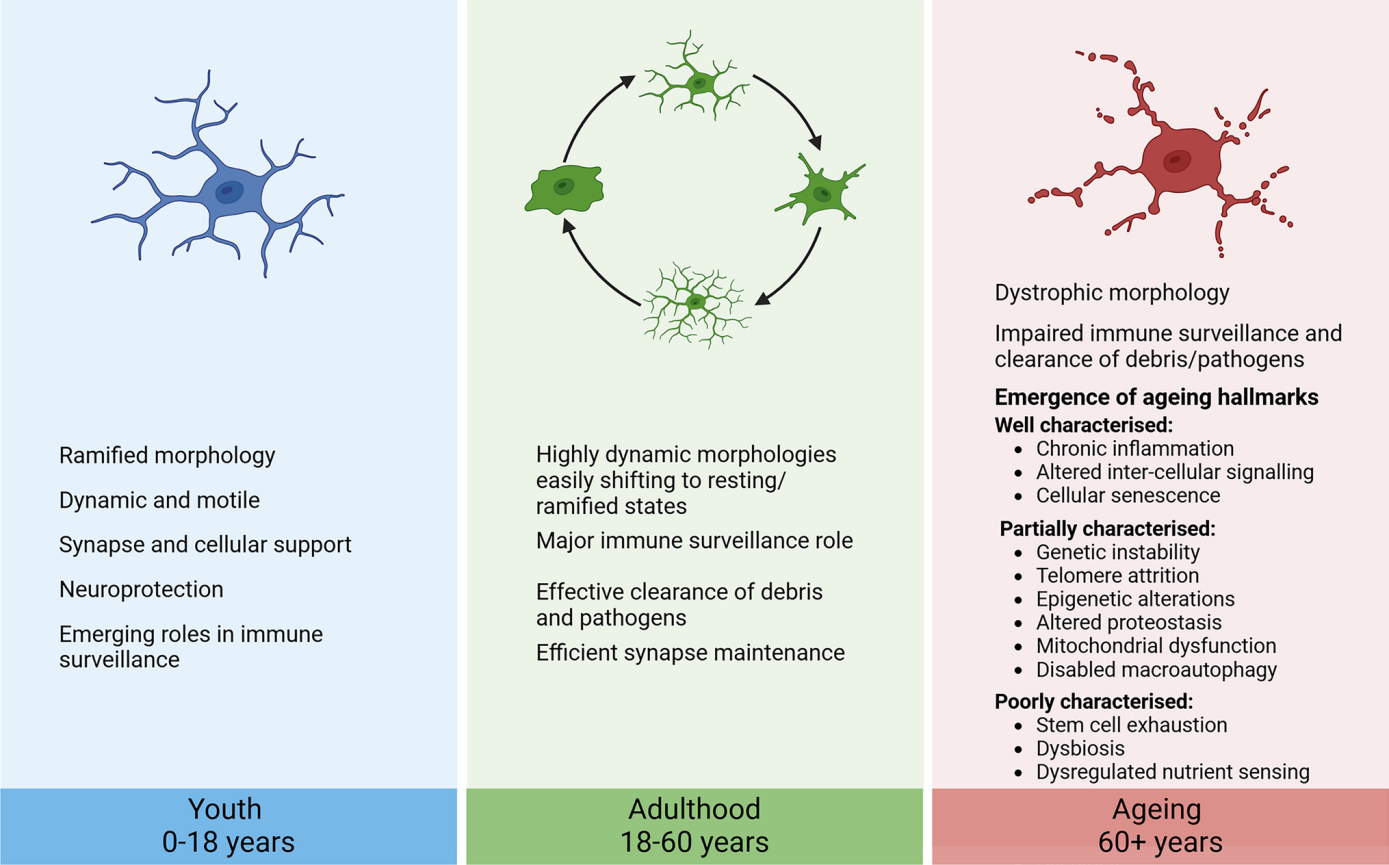

## Introduction

### Brain Changes with Age

As our population ages, healthy ageing and the ability to maintain an independent lifestyle in the later years of life are becoming increasingly relevant (Dlima et al. 2024). Globally, the number of individuals over the age of 60 is projected to double by 2050 (Rudnicka et al. 2020). This is significant, as ageing is the number one risk factor for developing neurodegenerative diseases (ND), such as Alzheimer’s Disease (AD) and Parkinson’s Disease (PD) (Hou et al. 2019), leading to dramatic expected increases in prevalence for these diseases (Hou et al. 2019; Pallas et al. 2008). In line with this, the number of global AD cases increased 160.84% from 1990–2019 (Nichols et al. 2019) and the number of PD cases rose 155.5% within the same period (Ou et al. 2021). Notably, both advanced age and NDs share alterations in several common underlying physiological processes, impacting the function of central nervous system (CNS) cells. López-Otín and colleagues (López-Otín et al. 2023) outlined 12 hallmarks of ageing, many of which show overlapping patterns of change with NDs. In fact, recent reviews have focused on these hallmarks in AD (Zhao and Huai 2023; Liu et al. 2024) and PD (Coleman and Martin 2022). Specifically, these 12 hallmarks include genomic instability, telomere attrition, epigenetic changes, loss of proteostasis, disabled macroautophagy, dysregulated nutrient sensing, mitochondrial dysfunction, cellular senescence, stem cell exhaustion, altered intercellular communication, chronic inflammation and dysbiosis. These are defined further in Table 1, with direction provided for further reading on the presentation of each of these in age-related NDs.

**Table 1:** Definitions of the 12 hallmarks of ageing and their presentation in NDs

Hallmark	Definition and main features as described by López-Otín and colleagues (López-Otín et al. 2023)	Presentation in age-related NDs	
		AD	PD
Genomic instability	The accumulation of genetic damage with age • Loss of DNA repair mechanisms • Accumulation of DNA damage • Increased frequency of DNA double-strand breaks	↑DNA damage and impaired DNA repair mechanisms in post-mortem AD brains Reviewed: (Hou et al. 2017)	↑DNA damage in post-mortem PD brains and experimental PD models Recent review: (Wang et al. 2023)
Telomere attrition	The progressive shortening of telomeres • ↓telomerase activation • ↓telomerase reverse transcriptase (TERT) and telomerase RNA component (TERC) • Loss of telomere maintenance mechanisms	↓peripheral telomere length in AD patients and telomere maintenance protects against AD pathology Recent review: (Kuan et al. 2023)	Conflicting reports on telomere dynamics in PD. Lengthening and shortening observed in leukocytes but no changes observed in substantia nigra Recent review: (Vellingiri et al. 2023) Meta-analysis showing no association between PD and telomere length: (Forero et al. 2016)
Epigenetic changes	Changes in epigenetic pathways • Histone modifications • DNA methylation • Chromatin remodelling • Altered gene expression without changes to DNA sequences	Post-mortem AD brains show ↑DNA methylation and abnormal localisation of histone modifications Recent review: (Gao et al. 2022)	Alterations in DNA methylation in human PD samples and experimental models Recent review: (Sharma et al. 2024)
Loss of proteostasis	De-regulation of protein homeostasis • ↑ oxidative protein damage • Failure of protein quality control • Misfolded and incomplete proteins • Changes in proteasome capacity	Ubiquitination and protein synthesis mechanism dysfunction is associated with AD risk and pathology in experimental AD models Recent review: (Cozachenco et al. 2023)	Proteasome components are inhibited by Lewy body pathology and loss of proteasome is associated with PD pathology in the substantia nigra Review: (Lehtonen et al. 2019)
Disabled macroautophagy	Impaired autophagic recycling of damaged organelles and proteins • ↓ recycling of cytoplasmic components	Dysfunction associated with AD pathology Recent review: (Liu and Li 2019)	Activation of macroautophagic pathways associated with neuronal degeneration and PD pathology Recent review: (Nechushtai et al. 2023)
Deregulated nutrient sensing	Dysregulation of metabolic pathways involved in nutrient sensing • ↑activity of mTOR pathway • Dysregulated Insulin sensing • ↓AMPK and sirtuin pathways	Signalling pathways are impaired in human AD and in experimental AD models mTOR pathway reviewed: (Perluigi et al. 2021) insulin pathways reviewed: (Gabbouj et al. 2019) AMPK pathway reviewed: (Cai et al. 2012)	Signalling pathways are impaired in human PD samples and in experimental PD models mTOR pathway reviewed: (Lan et al. 2017) Insulin pathway reviewed: (Castilla-Cortázar et al. 2020)
Mitochondrial dysfunction	Dysfunctional mitochondrial homeostasis and ↓function • ↓mitochondrial turnover • ↑ROS production • ↑mitochondrial membrane permeability	Mitochondrial damage and dysfunction present in experimental AD models Recent reviews: (Bhatia et al. 2022; Leuner et al. 2012)	Mitochondria in post-mortem PD tissue and experimental models show reduced activity and impaired function Recent review: (Henrich et al. 2023)
Cellular senescence	A state of irreversible growth arrest to prevent proliferation of damaged cells • Cell cycle arrest (↑p16^INK4A^, ↑ p21^Cip1/Waf1^) • Formation of senescence-associated heterochromatin foci (SAHF) • ↑ Inflammatory profile	Neuronal and glial senescence associated with AD pathology (Bussian et al. 2018; Herdy et al. 2022)(Bhat et al. 2012) Recent review: (Liu 2022)	Glial and neuronal senescence evident in experimental PD models and post-mortem PD brain tissue (Chinta et al. 2018; Shen et al. 2024) Recent review: (Miller et al. 2022)
Stem cell exhaustion	Decline in number and function of stem cells • ↓Tissue renewal/impaired repair	Altered neurogenesis (Haughey et al. 2002) AD pathology decreases proliferation in human neural stem cells (Lee et al. 2013)	MPTP induces a premature senescent phenotype human neural stem cells (Zhu et al. 2015)
Altered intercellular communication	Changes in signalling between cells • Altered activity of neural and hormonal pathways • Changes in extracellular molecules and cytokines • Changes in junction mediated communication	Alterations in neuronal signalling Reviewed: (Gadhave et al. 2021)	Alterations in substantia nigra dopaminergic signalling Recent review: (Dong-Chen et al. 2023)
Chronic inflammation	Low-grade, persistent inflammation • ↑inflammatory responses ↓immune function • Overexpression of pro-inflammatory cytokines • Activation of immune cells	↑inflammatory markers and glial activation in experimental and post-mortem brain tissue Recent review: (Wong-Guerra et al. 2023)	↑ peripheral inflammatory cytokines in PD patients ↑microglial activation in post-mortem tissue Recent review: (Tansey et al. 2022)
Dysbiosis	Imbalance of the gut microbiome • ↓microbial diversity and ↑harmful microbes	↓microbial diversity, ↑gut-brain axis dysregulation Recent review: (Liu et al. 2020)	Altered composition of gut microbiome Recent systematic review: (Li et al. 2023b)

The pathological commonalities between ageing hallmarks and NDs underscores the need to disentangle the independent effects of physiological age-related changes from those due to disease processes. Understanding each of these hallmarks on a molecular level is crucial for improving our ability to replicate the cellular environment that occurs in NDs. However, these changes may present differently in different CNS cell types. For example, cell cycle arrest is a defining characteristic of cellular senescence, a hallmark of ageing (López-Otín et al. 2023); however, in post-mitotic cells, such as neurons, cell cycle arrest does not function as a marker of an aged cell (Sapieha and Mallette 2018). Conversely, microglia and astrocytes are capable of division (Joya and Martín 2021) and, thus, have the potential to express this element of cellular senescence. Given the role of glial cells in supporting neuronal health (Rahman et al. 2022), characterising ageing in these support cells is critical. While both microglia and astrocytes have important immune functions in the CNS (see reviews (Borst et al. 2021), (Giovannoni and Quintana 2020)), microglia are considered the primary drivers of the immune response (Ginhoux et al. 2013) and are the focus of the current review.

## Microglial Function Across the Lifespan

**Fig. 1:**
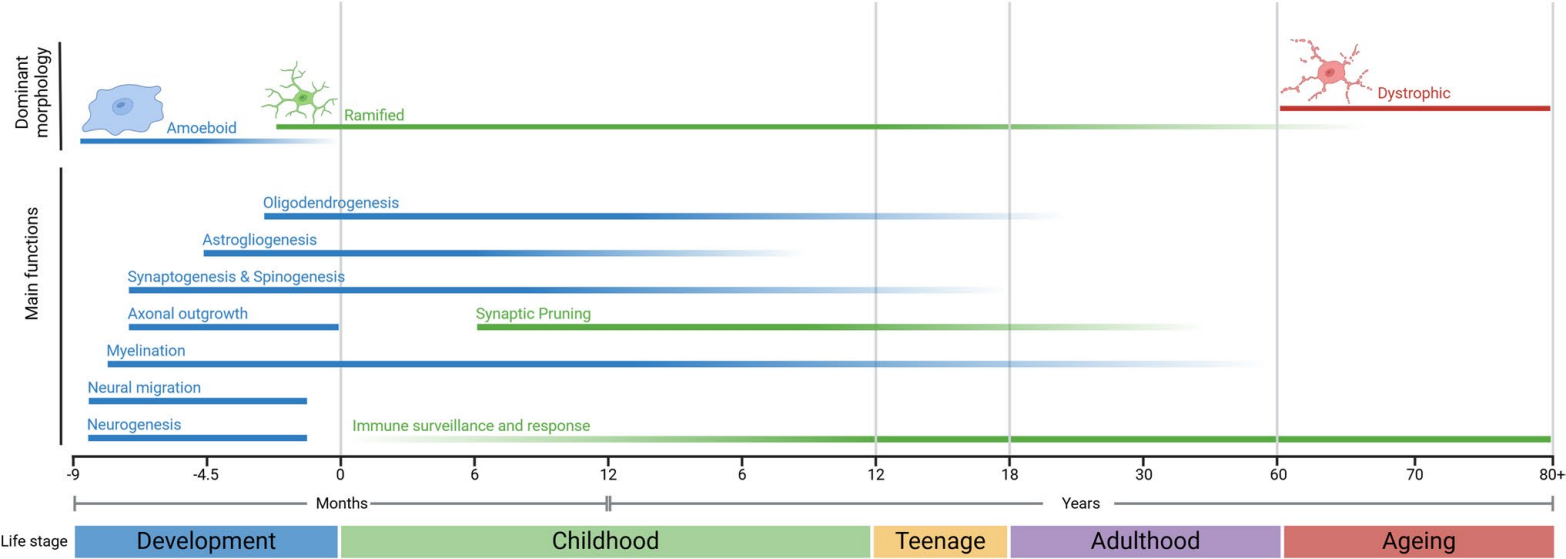
Microglial roles and morphologies during different stages of life. During development and early childhood, microglia have major roles in supporting the development of the CNS. Before birth, microglia display an amoeboid morphology. Ramified morphology begins to become the dominant morphology at the time of birth and remains so until late adulthood. During childhood, microglia maintain developmental roles through synaptic pruning, but transition to their major immune role around adolescence. During ageing, microglia acquire a dystrophic morphology; however, the functional changes at this stage remain unclear. Figure created in BioRender

Microglia are the tissue resident macrophages in the CNS, accounting for 5–20% of the total cell population in the brain (Hugh Perry 1998). Microglia arise from progenitor cells in the yolk sac during early development (Cuadros et al. 1993; Alliot et al. 1999), where they are responsible for facilitating cell migration, neurogenesis and gliogenesis (Menassa and Gomez-Nicola 2018). Conversely, while they have responsibilities in CNS maintenance, including synaptic pruning and phagocytosis of apoptotic cells (Borst et al. 2021), their major role in the adult CNS is the initiation of an adequate immune response to injury or infection (Ginhoux et al. 2013). Due to their dual role in both homeostasis and immune functions, microglia are highly dynamic cells, with constantly changing morphologies and functional phenotypes throughout the lifespan (Fig. 1).

During development and early childhood, microglia are amoeboid and migratory cells (Menassa et al. 2022), responsible for neurogenesis (Matsui and Mori 2018; Cunningham et al. 2013), neuronal migration (Ueno and Yamashita 2014), synaptic connections (Miyamoto et al. 2016) and axon growth (Squarzoni et al. 2014)). Additionally, microglia support the differentiation of astrocytes and oligodendrocytes from progenitor cells (Reemst et al. 2016; Borst et al. 2021). This ensures successful ongoing brain development and facilitates CNS plasticity during critical developmental periods (Mallya et al. 2019). Throughout the late teenage/young adult years, microglia further facilitate synaptic pruning to maintain network plasticity (Paolicelli et al. 2011; Mastenbroek et al. 2024). As individuals progress through early and middle adulthood, microglia maintain a surveillant non-inflammatory phenotype, with much of their population displaying ramified morphology (Harry and Kraft 2012).

Conversely, as individuals move into older adulthood (i.e. 60 + years of age), microglia typically display a dystrophic morphology (Greenwood and Brown 2021), also called “senescent microglia”, which features marked de-ramification with remaining processes fragmented or swollen (Neumann et al. 2023). These cells have been mainly characterised in aged human post-mortem tissue (Lopes et al. 2008; Streit et al. 2004) and studied to a lesser extent in aged mice (d'Avila et al. 2018; St-Pierre et al. 2022). Such microglial dystrophy has been proposed as more reflective of a disease-associated phenotype, rather than ageing itself (Streit et al. 2009; Shahidehpour et al. 2021). However, the idea that microglia develop an abnormal morphology, characterised mainly by process swelling and fragmentation, during ageing remains the general consensus (Wendimu and Hooks 2022; Paolicelli et al. 2022; Augusto-Oliveira et al. 2025). Beyond morphological change, it is well documented that microglia have a decreased ability to perform homeostatic functions and exhibit an altered immune response with advancing age (Niraula et al. 2017). This includes a more reactive phenotype, with microglia existing in a primed state featuring heightened immune responses, with longer time required to resolve the response following immune pathway activation (Norden et al. 2015). In line with this, when compared to microglia in 3-to 4-mo male Sprague–Dawley rats, those from 22-month-old (mo) rats showed higher numbers of microglia featuring an “activated” morphology (short, thick processes and small cell body) following intracerebral haemorrhage induced by collagenase injection to the striatum (Wasserman et al. 2008). Further, microglia from these aged rats demonstrated a prolonged inflammatory response following injury (Wasserman et al. 2008). Similarly, 18-to 20-mo male BALB/c mice demonstrated a hyperactive microglial response to lipopolysaccharide (LPS) stimulation compared to 3-to 4-mo mice (Henry et al. 2009).

To better understand microglial diversity, the literature has proposed various systems of classification and subtyping based on transcriptomic and proteomic analyses, as well as morphological distinctions. Transcriptomics provides a valuable tool to distinguish populations of microglia during ageing and disease (Stratoulias et al. 2019). This analysis has been used to identify specific subtypes of microglia using single-cell RNA sequencing in tissue collected from both human post-mortem samples and mouse AD models. Keren-Shual and colleagues (2017) identified a disease-associated microglia (DAM) subtype which exists in close proximity to markers of disease pathology and has a unique signature (Keren-Shaul et al. 2017). This signature is characterised by increased phagocytosis and activation of pathways linked to increased AD risk (including APOE, TREM-2 and CTSD) (Keren-Shaul et al. 2017). The DAM subtype has also been linked to other neurodegenerative conditions, including Amyotrophic Lateral Sclerosis (ALS) (Keren-Shaul et al. 2017; Jauregui et al. 2023) and PD (Schonhoff et al. 2023). Outside of disease, age-related transcriptomic profiles of microglia have also been identified using single-cell RNA sequencing. For example, Olah et al. (2018) identified a set of genes expressed primarily by microglia in the aged human dorsolateral prefrontal cortex, which was termed the HuMi_Aged dataset. When compared to another transcriptomic dataset from middle-aged microglia (Zhang et al. 2016), genes such as nuclear factor κ B subunit 1 (NFKB1), interleukin 1β (IL1 β), triggering receptor expressed on myeloid cells 1(TREM-1), Toll-like receptor 4 (TLR4) and cluster of differentiation 83 (CD83) were found to be downregulated with age. In contrast, genes including translocator protein (TSPO), cluster of differentiation 14 (CD14), presenilin enhancer gamma-secretase subunit (PEN-2), and cathepsin D (CTSD) were upregulated (Olah et al. 2018). This suggests that microglia undergo a shift in their genetic profile with age, involving alterations in immune signalling and responses. Further, transcriptional analysis of microglia across the mouse lifespan (E14 to P540) has been conducted by Hammond et al. (2019). This study did not identify specific clusters of microglia, but instead observed the expansion of inflammatory and interferon responding groups of microglia at 18-mo of age. Interestingly, however, despite identifying these enriched clusters, they made up only a small overall number of microglia, indicating that while numbers of pro-inflammatory cells may increase with age, the majority of the microglial population, at least in in mice, does not show a drastic transcriptomic shift with increased age (Hammond et al. 2019). This disparity between microglial changes in human and mice highlights the need for cross-species studies to understand the translatability of results, although a discussion of this is outside of the scope of the current review (for review see: (Barron et al. 2021)).

Similarly, proteomic analysis has contributed greatly to our understanding of microglia. Microglia isolated from the brains of patients undergoing treatment for medial temporal lobe epilepsy (*n* = 3) underwent proteomic analysis using cytometry by time of flight (CyTOF) (Böttcher et al. 2019). This analysis uncovered distinct patterns of protein expression across brain regions, namely an increased expression of markers of microglial activation (CD68, CD86, CD45, HLD-DR) in the thalamus and subventricular zone compared to the cerebellum, temporal lobe and frontal lobe. Further, this study confirmed the expression of proteins P2RY12, TMEM119, TREM2, EMR1 and CD64 as part of a signature that could be used to distinguish microglia from other myeloid cell types (Böttcher et al. 2019). In another study, proteomic analysis of microglia isolated from the brains of the APP NL-F mouse model of AD revealed upregulation of antigen presenting and interferon response proteins, and downregulation of proteins involved in homeostasis and cell migration, at 6- and 12-mo compared to WT controls. This study provides a snapshot not only of how microglial protein signatures change with disease, but also with age, as these alterations were not observed in this model at 3 mo (Sebastian Monasor et al. 2020). During ageing, the microglial proteome in mice shifts to feature decreased expression of homeostatic proteins (P2RY12 and TMEM119 (Grubman et al. 2021)) and increased expression of lysosomal proteins (LYZ (Grubman et al. 2021), HLA-A and CST7 (Keane et al. 2021)), proteins involved in phagocytosis (LPL (Keane et al. 2021)) and microglial priming (ITGAX, SPP1 (Keane et al. 2021)), and antigen presenting proteins (HLA-DQB1 and CD74 (Grubman et al. 2021)). Overall, the use of proteomics to understand microglial function across health and disease is limited compared to transcriptomics (Davis and Lloyd 2024). These technologies, particularly when applied at the single-cell level, offer powerful insights into how microglial phenotypes change with age and disease. However, while both methods are gaining popularity in the field, they are not yet widely applied, partially due to their high cost and need for specialised infrastructure (Williams et al. 2022). As a result, more traditional approaches remain commonly employed, although these methods are not without their limitations. For example, morphological classification of microglia is widespread, despite its inherent subjectivity (Young and Morrison 2018), and difficulties associated with directly comparing between studies due to inconsistent terminology (Paolicelli et al. 2022). Nevertheless, the use of morphological classification, in conjunction with immunohistochemical or immunofluorescent staining for functional markers, continues to be the most used method to assess microglial phenotypes in in vivo pre-clinical studies.

## Molecular Mechanisms and Morphology of Microglial Phenotypes

**Fig. 2:**
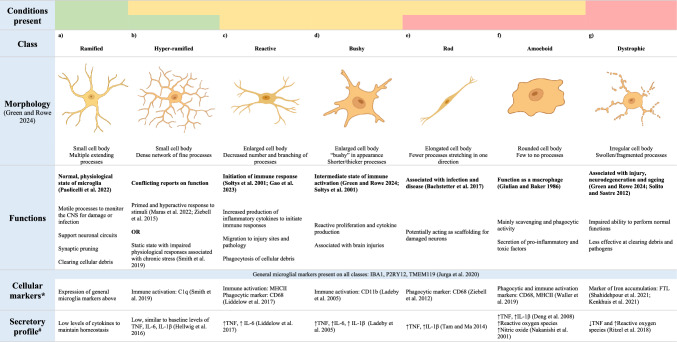
Microglial classifications used throughout the literature and associated morphologies, functions and markers. Green: Phenotypes present under physiological conditions, Yellow: Phenotypes present following induction of immune response (i.e. response to injury/infection), Red: Phenotypes present under dysregulated or pathological conditions a Ramified microglia are in a state of CNS surveillance, with low baseline levels of inflammatory cytokine release. **b** Hyper-ramified microglia functionally have either a heightened or an impaired response to stimuli. **c** Reactive microglia initiate the immune response. **d** Bushy microglia are thought to be transitioning towards immune activation and have some phagocytic and inflammatory functions. **e** Rod microglia are associated with degeneration and injury, with potential roles in structural support of neurons. **f** Amoeboid microglia function as macrophages, with main roles in phagocytosis and secretion of pro-inflammatory factors. **g** Dystrophic microglia are associated with neurodegeneration and ageing and have a diminished capacity to perform normal homeostatic functions. *Markers expressed on microglial cells within each classification. #Factors measured in conjunction with microglia for each classification. Images from BioRender

Various morphological classifications for microglia are utilised across the literature, which often have overlapping functions and markers. Studies have observed changes in cellular markers and secretory profiles, alongside shifts in microglial morphology, which has allowed researchers insight into potential functions of morphological phenotypes (e.g. rod microglia (Bachstetter et al. 2017)). These morphological classifications and observed corresponding functions, markers and secreted factors are summarised in Fig. 2. Importantly, although morphology is a common method of classifying microglia, morphological change does not always correlate with an overall shift in function. Therefore, when assessing changes in microglial populations, it is best practice to pair morphological analysis with the use of known functional markers, such as MHCII, to assess immune activation (Jurga et al. 2020). It should be further noted that microglial classification throughout the literature is inconsistent, with morphological terms being used interchangeably such as bushy and hyper-ramified (see: Ziebell et al. 2015; Reddaway et al. 2023)). Further, less characterised morphologies, such as rod microglia are vaguely described, with little understanding of their functional phenotype (Giordano et al. 2021).

**Fig. 3:**
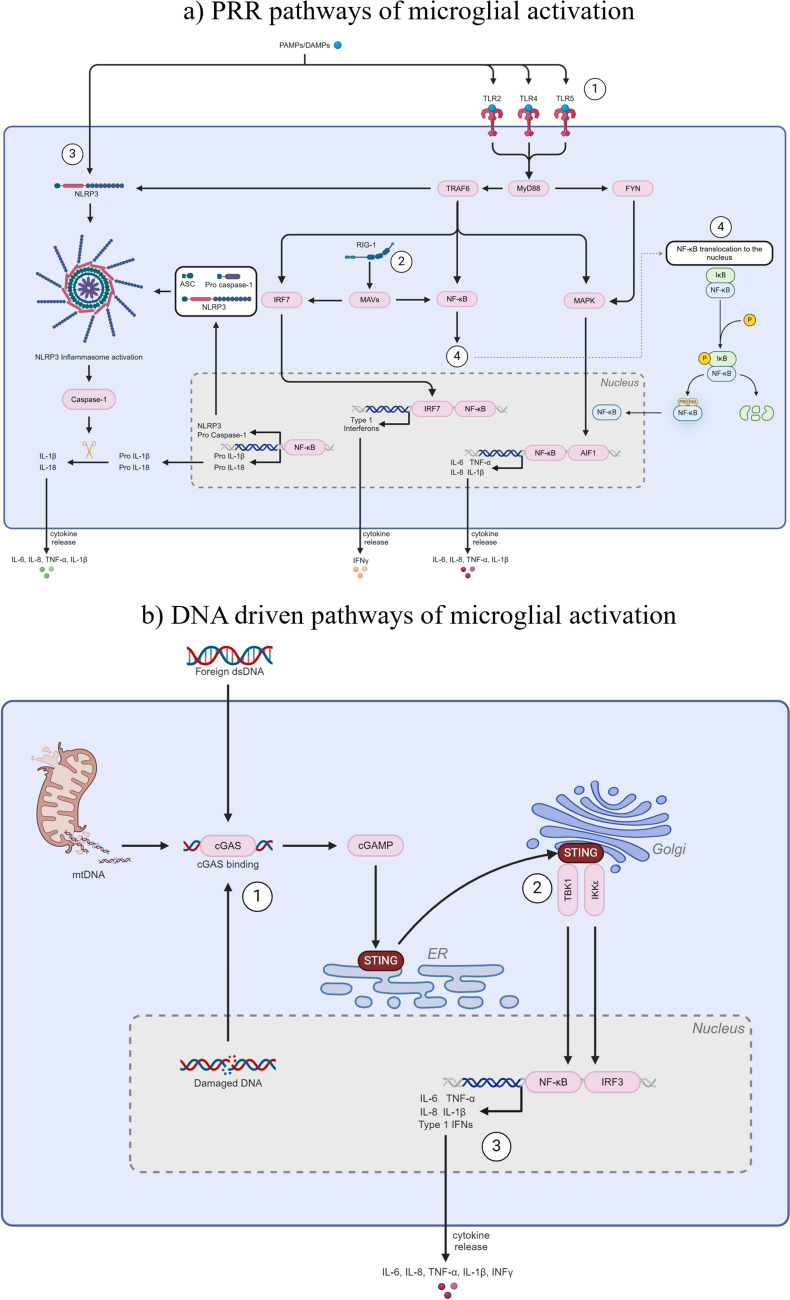
Pathways of microglial responses. **a** PRR response pathways in microglia. PRRs, such as 1. TLRs, 2. RLRs, and 3. NLRs, recognise PAMPs/DAMPs and trigger signalling cascades, including 4. Exposure of NF-κB subunits by degradation if IκB and translocation of NF-κB to the nucleus. These cascades cause the downstream release of inflammatory cytokines. **b** DNA-driven immune responses in microglia. 1. cGAS binds to free DNA in the cytosol (mtDNA, nuclear DNA, foreign DNA), which activates the cGAS-STING pathway, causing the transcription of pro-inflammatory genes and release of pro-inflammatory cytokines. Figure was created using BioRender

In response to injury or infection, the microglial immune response is initiated. This immune response involves alterations in expression of surface markers, secretion of signalling factors and is often accompanied by a shift in morphology. The classical definition of microglial activation is displaying a mobile, amoeboid morphology (Fig. 2f), increased phagocytosis and increased release of pro-inflammatory cytokines (Giulian and Baker 1986). The activation of a microglial immune response has often been shown to occur in the presence of protein aggregation, as occurs during ageing and in age-related NDs (Scheiblich et al. 2021; Barger and Harmon 1997; Gao et al. 2023). This activation occurs through a number of pathways involving pattern recognition receptors (PRRs), which allow the recognition of pathogenic signals such as damage-associated molecular patterns (DAMPs), and pathogen-associated molecular patterns (PAMPs) (Glezer et al. 2007; Kigerl et al. 2014). These pathways are summarised in Fig. 3a; however, microglia can also undergo inflammatory activation driven by intracellular DNA-sensing mechanisms which are summarised in Fig. 3b.

Historically, microglia have been referred to as resting or activated. However, these definitions are now considered inaccurate, as microglia are in fact constantly “active” and perform many functions under homeostatic conditions (Paolicelli et al. 2022). Notably, studies often use the term “microglial activation” to refer to microglia that are reacting to a stimulus or undergoing the induction of an immune response. The major phenotypes that this terminology encompasses include those that are present under homeostatic conditions, during an immune response, and under disease conditions. As shown in Fig. 2, various morphological classes of microglia can exist under both homeostatic and responsive states. When discussing previous publications where microglia have been referred to as activated, the current paper endeavours to specify the phenotype observed with reference to those outlined in Fig. 2. Where previous observations are vague or unclear, the terminology used by original authors has been used here, with additional information regarding the type of “activation” provided where possible.

Considering the aged background on which NDs such as AD and PD occur, it is reasonable to infer that age-related alterations in microglia are a major contributor to disease pathophysiology in NDs (Gamage et al. 2023; Zhang et al. 2023). Given this link, the importance of including age as a factor in the pre-clinical modelling of NDs cannot be understated. However, there is a clear lack of consideration of age in current pre-clinical studies of NDs (reviewed: Carr et al. 2024; Sun et al. 2020)). In order to better model the age-related changes occurring in CNS cells, there needs to be broader understanding of how the CNS immune response changes with advancing age (Angelova and Brown 2019), particularly the role of microglia in this process.

## Understanding Molecular Changes in Microglia Through the Hallmarks of Ageing

As discussed above, changes in microglial inflammatory pathways and morphology with ageing and disease have been previously described. However, there has been little characterisation of microglia with reference to the known hallmarks of ageing. The hallmarks of ageing (López-Otín et al. 2023) represent specific patterns of change in critical systems which can distinguish an aged phenotype and have been observed in both AD and PD (Table 1). These hallmarks have been described in peripheral macrophages (Guimarães et al. 2021) but are less well investigated specifically in microglia, with studies instead focusing on how microglial production and release of pro-inflammatory factors and microglial immune responses change with age. Below, we review the studies to date that have explored the presentation of these hallmarks in microglia with advancing age, with a focus on how these changes may be relevant in the context of neurodegeneration.

### Genomic Instability

Genomic instability as a hallmark of ageing is defined by an accumulation of damaged DNA and loss of or alterations in DNA repair pathways (Chen et al. 2022). In microglia, this has been studied both preclinically and in human post-mortem tissue. In 24-mo C57BL/6 male and female mice, microglia have been shown to accumulate damaged DNA fragments as identified by γ-H2A.X staining and an increase in the percentage of pSTING + /CD11b + cells compared to 2-mo animals (Arvanitaki et al. 2024). These changes indicate activation of the DNA-driven immune response as a contributing factor to the acquisition of a pro-inflammatory phenotype in microglia. Similarly, the *Ercc1*^*Δ/−*^ mouse is deficient in DNA repair mechanisms and displays features of accelerated ageing. In *Ercc1*^*Δ/−*^ mice at 16-weeks-old following peripheral LPS injection, microglia displayed hypertrophied processes and an increase in cell body size, along with increased proliferation, phagocytosis and ROS production compared to wild-type (WT) controls (Raj et al. 2014). In addition, *Ercc1*^*Δ/−*^mice showed evidence of microglial priming, indicated by an enhanced inflammatory response following peripheral LPS challenge, a result which has also been replicated in *Ercc1*^*Δ/−*^mice at older ages (4-, 8- and 14-mo) (Zhang et al. 2021). While DNA damage itself was not explicitly measured in microglia in either of these studies, these results implicate a loss of DNA repair mechanisms in driving microglial ageing and pro-inflammatory microglial responses.

Conversely, in human post-mortem tissue from 14 healthy controls (6M:8F) aged 65–96 years old, dystrophic microglia did not show positive γ-H2A.X staining (Neumann et al. 2023), but were strongly correlated with increased ferritin expression, a marker also known to increase with ageing. Ferritin protein, most prominently expressed in microglia in the brain, can store excess iron and increased expression of ferritin in microglia can be used as an indicator of iron overload in the brain (Lopes et al. 2008; Streit et al. 2018). Iron overload has been linked to increased γ-H2A.X in hiPSCs (Han et al. 2020) with the role of ferritin in microglia proposed to be to mediate the damaging effects of excess iron in the brain (Neumann et al. 2023). However, the accumulation of iron-containing ferritin protein has been suggested as a contributing factor to the dystrophic morphology observed in aged microglia (Neumann et al. 2023). While healthy control brains did not show increased DNA damage markers with age when assessed by γ-H2A.X staining, another human post-mortem study in older adults (mean age 94.07 years ± 0.95) identified a subset of genes preferentially expressed by aged microglia, termed the HuMi_Aged dataset (Olah et al. 2018). DNA damage-associated pathways were enriched in this dataset, along with telomere maintenance and chromosomal maintenance pathways (Olah et al. 2018). These results together suggest that while γ-H2A.X expression is not associated with aged microglia in humans, alterations in DNA damage pathways are indeed present in aged human microglia. However, the specific changes which occur due to alterations in these pathways are yet to be identified. These results should also be interpreted with caution, as they are derived from only two studies, with the former containing a relatively small sample size and no young control cohort. This represents a wider issue in the field, with young healthy brain tissue difficult to obtain (Bell et al. 2008), making human post-mortem studies challenging and leaving remaining questions about how well age-related changes observed in pre-clinical rodent models reflect those seen in the in human brain. While large animal models, which are arguably more translational (Sorby-Adams et al. 2018), can be used to investigate microglial alterations, to our knowledge, none have yet been used to investigate changes that occur in the microglial genome with age. This necessitates further research into this area to uncover the extent to which typical markers of senescence, such as accumulated DNA damage, can be observed in aged microglia across species.

### Telomere Shortening

Telomere shortening is one of the most widely recognised and accepted hallmarks of ageing (Fasching 2018). Telomeres, protective DNA sequences which cap the end of chromosomes, function to stabilise the ends of chromosomes and protect genomic material from degradation (Vaiserman and Krasnienkov 2021). However, with each cell division, telomeres become progressively shorter due to the inability of DNA polymerase to fully replicate the end of the C-rich lagging strand (Vaiserman and Krasnienkov 2021). This effect is referred to as the end replication problem, where telomeres will shorten until reaching a critical length where they can no longer protect the chromosome (Vaiserman and Krasnienkov 2021). This triggers cell cycle arrest and cells either enter a senescent state or undergo apoptosis. Telomere length has been widely utilised as a biomarker of ageing (Sanders and Newman 2013). However, there is debate as to whether these pre-clinical results have translational relevance for human ageing, where there have been conflicting results regarding the association of telomere length and age. While a full discussion of this debate is beyond the scope of this review, detailed information regarding the validity of telomere shortening as a marker of ageing can be found: (Mather et al. 2011; Vaiserman and Krasnienkov 2021).

The evidence for telomere length as a marker of ageing has come primarily from peripheral blood leukocytes and fibroblasts. A 2022 systematic review and meta-analysis of 27 studies reported that longer leukocyte telomeres were associated with lower levels of age-related structural change (brain volume loss) and cognitive decline in non-demented cases (Gampawar et al. 2022). Telomere alterations in the brain itself have been less well investigated. Nevertheless, some studies in isolated rat microglia demonstrate telomere shortening with age. The first study to investigate glial telomeres was conducted by Flanary and Streit (Flanary and Streit 2004), who found telomeres in microglia from neonatal rat cortices shorten over 32 days in culture when measured using flow cytometry fluorescent in situ hybridization (flow-FISH). Telomerase activity in these microglia declined steadily until day 24, but increased dramatically from then until the end of the 32-day culture period (Flanary and Streit 2004). Similarly, in a 2007 study by the same authors, microglia isolated from 30-mo male Fisher-344 rats were found to have shorter telomeres than microglia isolated from 3-mo rats when measured using flow-FISH (Flanary et al. 2007). These aged microglia also had decreased telomerase activity compared to young microglia, indicating both a decline in telomere length and telomere maintenance with age in rodent microglia (Flanary et al. 2007). Of note, the authors also investigated microglial telomere length in human post-mortem tissue from AD patients in this study. Microglia were isolated from the superior frontal gyrus/frontal pole region of 4 AD (86 years old ± 2.54) and one non-dementia case (86 years old), and microglial telomere length and telomerase activity were measured. Telomeres in AD microglia were significantly shorter than those seen in the non-dementia case; however, no changes in telomerase activity were observed, indicating that decreases in telomerase activity are not necessary for shortening of telomeres to occur (Flanary et al. 2007). Notably, however, there was no investigation of microglial telomere length compared to young human tissue, likely due to limitations associated with obtaining post-mortem tissue from young donors, as discussed above.

Several other studies have also investigated telomere shortening in the context of NDs. For example, one study utilised a cross between the APP23 transgenic mouse model of AD and the Telomerase RNA component (Terc) knockout model of telomere shortening (G3Terc^−/−^APP23^+^) to investigate AD pathology on the background of shortened telomeres (Rolyan et al. 2011). Interestingly, at 12-mo G3Terc^−/−^APP23^+^ mice had reduced Aβ pathology in the frontal cortex, as well as improved spatial memory compared to APP23^+^ mice. In addition, G3Terc^−/−^APP23^+^ mice showed no increase in microglial immune activation (MHCII^+^ microglia) in the vicinity of Aβ plaques (Rolyan et al. 2011). Similarly, a cross between a pre-clinical model of PD, the Thy-1[A30P] α-synuclein transgenic mouse model, and Terc^−/−^ mice (αSYN^tg/tg^G3Terc^−/−^) showed decreased microglial Il-1β gene expression and reductions in expression of MHCII in the brainstem of 17-mo αSYN^tg/tg^G3Terc^−/−^ mice compared to αSYN^tg/tg^ mice (Scheffold et al. 2016). Taken together, these results surprisingly seem to suggest that telomere shortening is protective against the induction of microglial immune responses to disease pathology. However, contrary to what was observed in the AD mouse model, accelerated disease phenotype and early death were seen in PD mice. This suggests that loss of telomere maintenance may inhibit a protective response by microglia, ultimately leading to worsened disease phenotypes, at least in older animals. Further research is required to unravel the mechanisms that underlie these processes. In addition, as telomere shortening was artificially induced via Terc knockout in both of these previous studies, it is important to investigate how this may differ from changes that occur with physiological ageing.

### Epigenetic Changes

Epigenetic changes encompass a range of heritable changes that cause alterations in gene expression, without directly changing DNA sequences (la Torre et al. 2023). The changes most studied with regard to ageing include chromatin remodelling, histone modifications and DNA methylation (see review: (la Torre et al. 2023)). Alterations in all these processes have been reported to occur in microglia with advancing age, with DNA methylation and histone deacetylase pathways identified as two of the enriched pathways in the HiMi_Aged dataset discussed above (Olah et al. 2018), suggesting specific changes in these pathways in human-aged microglia. Chromatin remodelling refers to alterations in the structure and organisation of chromatin and includes altered chromatin accessibility (CA), a measure of how easily genes may be accessed for transcription (Li et al. 2023a). One study, which conducted mapping of CA by ATAC-seq in the microglia of female and male C57BL/6J mice at 3-, 14- and 24-mo, described a number of genes differentially expressed with age that were termed age-dependent microglia genes (ADEM genes) (Li et al. 2023a). Genes which positively correlated with age were termed P-ADEM genes (notably genes related to phagocytosis, IFN signalling, ROS production and antigen presentation), while genes that negatively correlated with age were designated as N-ADEM genes (including chemokine suppression and iron transportation genes). P-ADEM genes had a higher CA than N-ADEM genes in promoter regions, indicating that alterations in CA of ADEM genes may dictate the emergence of specific microglial phenotypes during ageing (Li et al. 2023a). For example, the increased CA of ROS production genes (*Cybb* and *Hp*) and IFN signalling genes (*Ifitm3, Ifi204, Cxcl16, Xaf1, Gas6* and *Tgtp2)* suggests a shift towards a more pro-inflammatory phenotype in microglia, potentially leading to increased microglia driven inflammation with age.

Histone modifications have also been shown to occur in microglia with advancing age. In line with this, an age-related decrease in the histone demethylase Jumonji domain-containing protein-3 (JMJD3) has been observed in microglia of male C57BL/6 mice aged 16–18-mo compared to 2- to 3-mo mice (Tang et al. 2014). JMJD3 is suggested to be necessary for microglia to initiate an anti-inflammatory response (Tang et al. 2014). Thus, its decrease with age is potentially representative of a shift towards a pro-inflammatory phenotype with advancing age. This study also found an increased level of the histone H3K27me3 in the midbrain of aged mice. H3K27me3 is the trimethylation of lysine 27 on histone H3, a histone modification which is associated with reduced transcriptional activity, which can be reduced by JMJD3. Thus, this increase in H3K27me3 may be a consequence of the decrease in JMJD3 seen in microglia with advanced age and may further contribute to imbalances in microglial responses.

Finally, DNA methylation refers to the addition of a methyl group to a DNA molecule, typically associated with silencing of genes. In microglia isolated from young adult (4- to 6-mo) and aged (24- to 26-mo) C57BL/6 mice exposed to LPS, an increase in IL-1β expression was observed with age (Matt et al. 2016). When probed further, IL-1β promoter DNA in aged microglia from older animals showed decreased methylation compared to that seen in younger animals (Matt et al. 2016). This suggests that, with age, silencing of pro-inflammatory cytokine genes is reduced following an inflammatory stimulus, potentially leading to an overactive microglial response. Overall, changes in CA and DNA methylation in microglia suggest a shift towards pro-inflammatory secretory phenotype, with histone modifications contributing to a reduced likelihood of taking on an anti-inflammatory phenotype. Taken together, this provides compelling evidence that microglia undergo a number of epigenetic changes with advancing age which cause an enhanced pro-inflammatory response. This response may be further exacerbated in the presence of ND-associated pathological proteins, such as Aβ in AD or α-synuclein in PD.

Overall, characterisation of epigenetic changes in microglia during ageing is sparce and specific pathways should be further investigated to understand the functional consequences of these changes. For example, Olah et al. (2018) identified DNA methylation and histone deacetylases as enriched in aged human microglia. Notably, however, this study did not investigate the specifics of either of these modifications and key questions remain. What genes are affected by the altered DNA methylation? Does this preferentially impact functional pathways in microglia, such as protein degradation or inflammatory pathways? Do either of these epigenetic alterations contribute to changes in overall microglial phenotypes with age? Investigating the functional consequences of these changes in microglia will improve our understanding of microglial ageing and which changes can be specifically targeted to improve outcomes with age and in NDs.

### Loss of Proteostasis

López-Otín and colleagues describe the hallmark loss of proteostasis as dysregulation of the intracellular mechanisms responsible for the degradation of proteins (López-Otín et al. 2023). A decline of proteostasis is often related to the aggregation and accumulation of proteins, a hallmark of neurodegenerative diseases such as AD and PD. Thus, the study of microglial proteostasis with age is critical in order to understand the mechanisms by which pathology may be targeted. In the HuMi_Aged dataset, the ER-phagosome pathway was enriched and upregulated in aged microglia (Olah et al. 2018) when compared to previous data generated from middle-aged microglia (Olah et al. 2018; Zhang et al. 2016). The ER-phagosome pathway facilitates communication of the endoplasmic reticulum (ER) with phagosomes and supports the maturation of phagosomes (Ghavami and Fairn 2022). This enrichment may, therefore, be reflective of a shift towards increased immune surveillance and increased antigen presentation. Indeed, Olah et al. (2018) also found enrichment of antigen processing-cross presenting pathways in their analysis. In addition, this enhanced activity of the ER-phagosome pathway may also be acting as a compensatory mechanism to combat proteostatic stress as a result of accumulation of misfolded proteins in the ER such as collagen 1 (De Leonibus et al. 2024), but no studies have yet investigated this mechanism in microglia. Further, proteomic analysis of microglia isolated from young (3- to 5-mo) and aged (20- to 24-mo) mice revealed differential expression in a number of proteins, including downregulation of proteasome complex subunits PSMB4, PSMA6 and PSMC1 (Flowers et al. 2017). Downregulation of these subunits would likely impair the ability of microglia to degrade damaged or misfolded proteins, leading to ineffective protein clearance in aged tissues. In AD, for example, microglia are important for degradation of Aβ (Qiu et al. 1997). However, if their capacity for proteasomal degradation is impaired, this may lead to accumulation of pathological Aβ within microglia, further disrupting proteostasis and protein degradation ability (Pomilio et al. 2020). In APP/PS1 mice also deficient in the immunoproteasome component LMP7, microglial immunoreactivity was decreased at 8-mo when compared to APP/PS1 mice with LMP7 of the same age. Interestingly, however, this change in microglial state did not alter the presence of Aβ pathology in these mice at any age (Wagner et al. 2017). It is important to note that this was only assessed at 8-mo, relatively young compared to other studies on microglial ageing, and results may, therefore, differ in older animals. Accordingly, tamoxifen-induced deletion of COP1 E3 ubiquitin ligase at ~ 2-mo of age resulted in increased expression of microglial genes involved in IFN signalling and APOE, a major AD risk factor gene, at 22-mo transgenic C57BL/6N mice, compared to WT mice (Ndoja et al. 2020). This result suggests that, in addition to accumulation of damaged or misfolded proteins, the loss of negative regulatory mechanisms, such as COP1 mediated ubiquitination, can promote pro-inflammatory signalling within microglia, which may contribute to sustained microglial immune activation during ageing.

Such effects may also be sex specific, at least to some extent. For example, in 18-mo-old C57BL/6 mice, microglia showed sex-dependent alterations in proteostasis. Microglia in female mice demonstrated a decreased ability to phagocytose Aβ42, while microglia from male mice showed a decrease in proteolytic gene expression, but no change in phagocytic abilities (Thomas et al. 2022). Of note, microglia from both sexes at 22-mo of age had decreased expression of the phagocytic receptor TREM2 (Thomas et al. 2022), suggesting a potential alteration in recognition of pathological proteins in aged microglia. Overall, there is mounting evidence that there is a de-regulation of the protein degradation pathways within microglia with advancing age. This may have significant implications for the pathogenesis of diseases like AD and PD, where an inability of microglia to combat pathological protein aggregation may contribute to the spread of pathology throughout the brain (Gao et al. 2023).

### Disabled Macroautophagy

Macroautophagy is defined as the recycling of large cargo and cellular contents by lysosomes, with cargo packaged into specialised vesicles called autophagosomes (Nieto-Torres and Hansen 2021). With age, the efficiency of cellular autophagy declines, resulting in a decreased turnover of organelles, protein and lipid degradation and reduced ability to dispose of pathogens (Nieto-Torres and Hansen 2021). In microglia, age-related autophagic changes are understudied, with most research to date focused on these changes in neurons. However, there has been some investigation of changes with age in microglial degradation pathways. Disruptions in macroautophagy have been investigated in vitro via knockout of ATG7, a key autophagic gene, in both primary microglia and BV2-microglia-like cells. ATG7 knockout has been shown to increase expression of Nod-Like Receptor Pyrin Domain-3 (NLRP3) inflammasome components CASP-1 and ASC following administration and uptake of Aβ (Cho et al. 2014). Thus, disruption of microglial autophagy may cause expression of pro-inflammatory factors and priming of microglia for expression of the NLRP3 inflammasome upon NLRP3 activation.

Although this mechanism has not been specifically investigated in the context ageing, disruption of Aβ receptor turnover in microglia from aged animals, combined with evidence of macroautophagic disruption in peripheral macrophages, strengthens the possibility that dysregulated receptor turnover occurs with age and impairs microglial responses. For example, in bone marrow derived macrophages isolated from 23-mo C57BL/6 mice, there was reduced expression of autophagic genes (ATG6 and ATG7) and impaired autophagic flux measured by LC3 (Stranks et al. 2015). Of note, like the above results seen in ATG7 deficient primary microglia, ATG7 deficient macrophages in this study demonstrated a pro-inflammatory phenotype. This suggests a role of dysregulated autophagy in macrophage-driven inflammation and solidifies the theory that similar processes may occur in microglia with age, potentially predisposing these cells to an inadequate response to ND pathology.

While distinct from macroautophagy, microglia also rely on distinct endocytic pathways to process extracellular material, and these pathways have been shown to change with age. LC3-associated endocytosis (LANDO) is critical in the turnover of Aβ receptors (including TLR4 and TREM2) on microglia, ensuring correct recognition of the Aβ protein (Heckmann et al.). Microglia isolated from 24-mo mice lacking the WD domain of Atg16L, which is necessary for LANDO recycling of Aβ receptors, demonstrated decreases in uptake of Aβ in culture compared to aged-matched WT controls, suggesting a role for Aβ receptor turnover in dysfunctional proteostasis with age (Heckmann et al. 2020). This suggests that turnover of microglial surface receptors is crucial to ensure that microglia can respond adequately to pathological proteins, and loss of these mechanisms with age may limit the ability of microglia to respond to pathology. Further investigation is necessary to determine whether macroautophagic and endocytic pathways interact in aged microglia and how potential changes in these pathways may influence microglial ageing.

### Dysregulated Nutrient Sensing

Ageing is associated with an overall decline in the availability of nutrients, due, in large part, to alterations in food and nutrition habits of older adults, influenced by reduced appetite, difficulties accessing food and altered taste and smell (Robinson 2018). Deficiencies in vitamin B12, Calcium, Iron and Vitamin D are common, as older adults rely more on heavily processed foods with low nutritional value (Rémond et al. 2015). In addition, changes in metabolism including insulin resistance and reduced mitochondrial efficiency, can influence nutrient processing at a cellular level (Gao et al. 2014). In response to these changes, key nutrient signalling pathways are affected, including the mechanistic target of rapamycin (mTOR), the 5’AMP-activated protein kinase (AMPK) (Jewell and Guan 2013; Khan et al. 2025) and insulin/Insulin-like growth factor-1 (IGF-1) signalling pathways. These disruptions affect cellular homeostasis, insulin responses and energy production across multiple tissues, including the brain, where these pathways and associated signalling cascades are critical in maintaining CNS homeostasis (Doust et al. 2022). The critical role of microglial metabolic plasticity in maintaining immune function under energetic stress has been highlighted by Bernier et al. (2020), who demonstrated that microglia from C57BL/6 mice (4-mo) are capable of rapidly adapting to glucose deprivation by switching to glutaminolysis (Bernier et al. 2020). This switch was reliant on mTOR-dependent signalling, highlighting the importance of effective nutrient sensing pathways in maintaining microglial function under stressful environments, such as are present during ageing and disease. In fact, the capacity of microglia to sustain bioenergetic processes has been shown to decrease with age. Compared to microglia from young mice (2–4-mo), microglia from aged mice (20–24-mo) demonstrate increased glucose conversion to glycogen and reduced mitochondrial respiration (Minhas et al. 2021), resulting in an energy deficient state. Importantly, this state was able to be reversed in microglia by inhibition of EP2 signalling, which restored metabolic function, suggesting that impairments in microglial nutrient sensing deficiencies are potentially targetable through metabolic reprogramming (Minhas et al. 2021). These results suggest that nutrient availability and metabolic flexibility in microglia are crucial for maintaining CNS homeostasis, but can become compromised during ageing, where dietary habits shift and nutrient deficiencies are common.

Changes in dietary habits can have profound consequences on microglial function specifically. For example, insulin resistance, which is commonly observed in older adults (Kolb et al. 2023), is hypothesised to drive microglia towards a pro-inflammatory state by the induction of hyperinsulinemia (Doust et al. 2022). The resulting increase in cytokine production may further inhibit insulin signalling in the brain. Interestingly, however, a previous study showed that microglia in the hippocampus of 22-mo Wistar rats did not mount an immune response following intracerebroventricular insulin administration for 5 days (Haas et al. 2020). Comparatively, increased numbers of IBA1 and CD68 positive microglia, as well as increased expression of COX-2 and IL-1β, were observed in 3-mo rats in response to the same treatment protocol within that study. This suggests that during ageing, the responsiveness of microglia to insulin signalling is diminished.

Interestingly, insulin/IGF-1 signalling has been linked with longevity in multiple species, including *C. elegans, Drosophila melanogaster* and laboratory mouse strains (see review: (van Heemst 2010)). In aged APP_SWE_/PS1_ΔE9_ mice (24-mo), a model of AD, IGF-1 mRNA and TNF mRNA levels were both increased in the hippocampus compared to age-matched WT mice. The authors theorised that this was due to an increase in IGF-1 mRNA-expressing microglia in the subgranular zone of the dentate gyrus (Myhre et al. 2019), but this was somewhat speculative. This would suggest that increased IGF-1 signalling is a key component of microglial pro-inflammatory responses in AD during advanced ageing, but comparison to microglia in young animals is needed to confirm this conclusion.

Another signalling pathway, the mTOR pathway, acts as a cellular energy sensor, responding to amino acids, glucose, oxygen and growth factors, including IGF-1 (Garza-Lombó et al. 2018). Dysregulated mTOR signalling with age, perhaps stemming from persistently elevated levels of IGF-1 or other growth factors, can lead to overactive nutrient sensing and contribute to the chronic, low-grade inflammation observed during ageing (reviewed: (Stallone et al. 2019)). This was observed in C57BL/6J mice, where mTOR activation was significantly increased at 23-mo of age compared to 6-mo of age in microglia isolated from female mice, as measured using phospho-flow cytometry (Keane et al. 2021). Studies in model organisms, such as *Saccharomyces cerevisiae* and *C. elegans,* have also supported the link between mTOR activity and ageing, where reduced mTOR activity has been shown to extend the lifespan (Fabrizio et al. 2001; Vellai et al. 2003). Despite this, studies investigating alterations in the mTOR pathway in aged microglia in these model organisms remain sparce.

The AMPK pathway serves as a central regulator of energy homeostasis, maintaining balance by counteracting mTOR activity (Garza-Lombó et al. 2018). AMPK responds to energy depletion (low ATP) by inhibiting anabolic processes and activating catabolic pathways (Garza-Lombó et al. 2018). These functions highlight the role of AMPK as a critical controller of ageing processes by modulating metabolism, oxidative stress and inflammation (Salminen and Kaarniranta 2012). Activity of the AMPK pathway has been shown to increase with age in the brain, with phosphorylation of AMPK in young male C57BL/6 mice (2- to 3-mo) lower than that seen in aged 16- to 18-mo mice, suggesting more AMPK activity in the brain during healthy ageing (Liu et al. 2012). However, in this same study, activation of AMPK following reversible middle cerebral artery occlusion was decreased in aged mice compared to young mice (Liu et al. 2012), indicating altered responsiveness of AMPK during ageing, potentially contributing to worsened outcomes following injury or pathology. Interestingly, the anti-inflammatory effects of AMPK activation in microglia have been previously demonstrated. Specifically, activation of the AMPK pathway by administration of 80µM of ENERGI-F704, a small molecule drug which causes phosphorylation and activation of AMPK, reduced the pro-Il-6 and TNF response of BV2 cells following LPS stimulation (Chen et al. 2014). This suggests an ameliorating effect, and potentially beneficial role of this pathway, in reducing microglial inflammation (Chen et al. 2014). Despite this, however, it is not yet known if and how levels of AMPK pathway activation are altered with age in microglia.

In order to understand the full effect of nutrient pathways on microglia in ageing, future research should seek to characterise changes in the responsiveness of microglia to nutrients and what downstream pathways are impacted by these changes. The apparent impact of insulin resistance on microglial responsiveness, coupled with evidence of altered IGF-1 signalling in ageing and AD, highlights a potential area of investigation for future studies. Profiling microglial expression of components of the insulin/IGF-1 pathway during ageing, potentially using single-cell transcriptomics, will increase our understanding of what mechanisms may drive these functional changes. Additionally, investigating other pathways in these cells, such as AMPK and its major components (e.g. mTOR and LKB1), will further elucidate the impact of nutrient sensing on microglial functions with ageing.

### Mitochondrial Dysfunction

Mitochondria are responsible for regulating a host of different cellular processes but are mainly involved in energy production and oxidative balance. They are a critical component of the free radical theory of ageing, due to their high production of and vulnerability to ROS. Additionally, damaged or dysfunctional mitochondria are involved in inflammatory processes, particularly by activation of the DNA-driven immune response by mtDNA damage or by free mtDNA released from damaged mitochondria (Fig. 3b). With age, mitochondrial function declines, leading to increased production of ROS and ROS-induced inflammatory responses (Amorim et al. 2022). Additionally, deficiencies in nutrient availability, as outlined in the above section, can have profound effects on mitochondrial metabolism and energy production. For example, Minhas et al. (2021) observed that altered glucose handling by PGE2-EP2 signalling in aged microglia shifted glucose towards glycogen storage rather than mitochondrial use, resulting in decreased mitochondrial respiration. Additionally, vitamin deficiencies inhibit the metabolic functioning of mitochondria and contribute to observed mitochondrial decay during ageing (Ames et al. 2005). Due to the high energy requirement for continuous immune functioning, microglia heavily rely on mitochondrial energy for maintaining function (Fairley et al. 2021) and are greatly affected by mitochondrial impairment and dysfunction (Ryu et al. 2003).

Mitochondrial transcription factor A (TFAM) is a promotor of mtDNA transcription and is critical in maintaining correct mtDNA transcription and regulating degradation of damaged mtDNA (Xu et al. 2019). A prior study investigated changes in this with age through overexpression of TFAM in male C57BL/6 mice at 2-mo and 24-mo. In this study, oxidative stress markers 8-oxo-deoxyguanosin (8-oxo-dG) and hydroxy-2-noneal (HNE) increased with age in microglia from WT mice, whereas no changes were reported in microglia from TFAM-overexpressing mice (Hayashi et al. 2008). This suggests that TFAM may play a protective role in maintaining mitochondrial and oxidative homeostasis in ageing microglia. Additionally, this study assessed whole brain malondialdehyde (MDA) levels, a marker of lipid peroxidation, and mitochondrial complex 1 activity (Hayashi et al. 2008). Aged WT mice demonstrated an increase in MDA levels, coupled with a decrease in complex 1 activity with age, suggesting age-related mitochondrial dysfunction and heightened oxidative stress. In contrast, TFAM-overexpressing mice showed no changes with age in either measure. While not specifically assessed in microglia, the increased 8-oxo-dG and HNE in aged microglia in these tissues suggest that microglial TFAM dysregulation may contribute to a broader oxidative stress observed in the ageing brain (Hayashi et al. 2008).

As well as damaged mtDNA accumulation with age, mtDNA can also move into the cytosol, caused during ageing by altered mitophagy, leading to the escape of both damaged and undamaged mtDNA from mitochondria and stimulating the DNA-driven immune response (Pérez-Treviño et al. 2020). In line with this, microglia from 20-mo C57BL/6J mice have shown an increased amount of cytosolic mtDNA compared to microglia from 2- to 3-mo mice (Gulen et al. 2023). This stimulated the cGAS-STING pathway in these animals, leading to the acquisition of a pro-inflammatory phenotype (Gulen et al. 2023). Taken together, these studies suggest that, with age, a loss of TFAM control of mtDNA leads to dysregulated ROS production, with dysfunctional mitophagy further contributing to the accumulation of mtDNA within the cytosol of microglia. Combined, these mechanisms increase the pro-inflammatory nature of aged microglia and increased ROS production likely causes further damage to mitochondria, feeding the cycle of continued inflammation and oxidative stress which is characteristic of both ageing and NDs. To date, however, limited studies have sought to characterise age-related changes in specific aspects of mitochondrial biology in microglia, with this an important avenue for future research.

### Cellular Senescence

Cellular senescence is the term used to refer to cells which have undergone an irreversible cell cycle arrest (Gorgoulis et al. 2019) and is commonly associated with ageing, as senescent cells are known to accumulate in aged tissues (López-Otín et al. 2023). There are many features of senescence (reviewed: (González-Gualda et al. 2021)), but the characteristic cell cycle arrest is the most defining feature. Indeed, microglia demonstrate changes in the senescence-associated markers of cell cycle arrest, p16^INK4A^ and p21^Cip1/Waf1^, with advancing age. In 18-mo male C57BL/6 mice, expression of p16^INK4A^, but not p21^Cip1/Waf1^, was observed in microglia (Ritzel et al. 2019). This study also reported an increase in DNA damage (H2A.X stain) and lipofuscin accumulation in microglia in these mice. Lipofuscin progressively accumulates within aged cells and is considered to be involved in a number of age-related pathologies (Jung et al. 2007). Indeed, an increase in lipofuscin accumulation has been demonstrated within microglia in 24-mo C57BL/6J mice of both sexes, although this was low in comparison to the levels that accumulated within neurons (Stillman et al. 2023). This indicates that while microglia do exhibit age-associated buildup of lipofuscin, they may possess more efficient clearance mechanisms than neurons; however, this still appears to become impaired with age.

Lipofuscin accumulation is often investigated in conjunction with senescence-associated β-Galactosidase (SA-β-Gal), as the presence of both has been shown to be related to lysosomal dysfunction with age (Georgakopoulou et al. 2013). In microglia, SA-β-Gal has been utilised to identify senescent microglia for further analysis. For example, to assess the proximity of senescent microglia to Aβ plaques, microglia in 8-mo 5XFAD mice (a model of AD pathology) and C57BL/6J WT mice were compared. Sagittal brain sections were stained for SA-β-Gal before undergoing 3,3’-diaminobenzidine (DAB) staining with IBA1 for microglial detection. Co-localisation of DAB (IBA1) with SA-β-Gal pigment demonstrated that the microglia surrounding pathological plaques were SA-β-Gal positive (Shin et al. 2024). SA-β-Gal staining has also been used to identify senescent microglia in vitro, identifying senescence in BV2 microglia-like cells following three or six treatments with the endotoxin LPS (Yu et al. 2012). Further, these BV2 cells demonstrated the acquisition of another well described marker of senescence, senescence-associated heterochromatin foci (SAHF). SAHF are regions of highly condensed chromatin formed in response to stresses, such as DNA damage and telomere dysfunction (Aird and Zhang 2013). These formations are seen in LPS-induced ageing in BV2 cells (Yu et al. 2012; Borgonetti and Galeotti 2023), but have not been assessed in in vivo studies looking at microglia change with advancing age.

Another marker of cellular senescence previously assessed in microglia is the acquisition of the senescence-associated secretory phenotype (SASP), characterised by an increase in the production and release of pro-inflammatory cytokines and chemokines (Rim et al. 2024). Microglia are known for their increased secretion of pro-inflammatory factors in NDs and indeed take on a similar phenotype during normal ageing. Microglia in 18-mo transgenic p7.2*fms-*EGFP mice on a C57BL6/6XCBA background have shown an increased release of pro- (IL-6, TNF, IL-1β) and anti- (IL-10) inflammatory cytokines compared to mice at 2-mo of age (Sierra et al. 2007). Additionally, microglia from the spinal cords of C57BL/6J male mice at 22-mo of age show an increase in inflammatory cytokine release (TNF, IL-1β), coupled with increased ROS-induced oxidative stress and microglial phagocytic ability (Ritzel et al. 2015). However, microglia are inherently inflammatory cells and many of the secreted factors associated with the SASP overlap with those secreted by microglia during the immune response (Ng et al. 2023). Additionally, as these cells are often studied in the context of diseases with an inflammatory component, using the SASP as a single marker of senescence in these cells is discouraged.

Across the literature, a stringent panel of markers for cellular senescence is not uniformly applied and many studies rely heavily on only one or two markers to distinguish senescent cells. This is problematic, as markers such as SA-β-Gal, the SASP and cell cycle inhibitors (p16 and p21) are not always specific to ageing, and often appear upregulated in other conditions (Ng et al. 2023). This could lead to the mislabelling of populations of cells as senescent. Issues with the definition of senescence as it relates to microglia have been previously discussed by (Ng et al. 2023), with a rigorous approach using multiple markers encouraged to define populations of senescent microglia. This integrated approach has been utilised by Hartmann et al. (2023) when establishing a measure of age in cultured fibroblasts. This approach examined telomere length, cell cycle inhibitors, SA-β-Gal and secretion of cytokines to create a scored age for cells in culture marked against actual chronological age of the donor (Hartmann et al. 2023). Future studies should endeavour to use a similar approach when defining senescence in microglia.

### Stem Cell Exhaustion

As a hallmark of ageing, stem cell exhaustion describes the decline in regenerative potential of adult stem cells. This is defined by López-Otín and colleagues (López-Otín et al. 2013, 2023) as the reduction of cell cycle activity of hematopoietic stem cells, contributing to the lack of capacity for tissue regeneration with age. Microglia originate from yolk sac progenitor cells (Cuadros et al. 1993; Alliot et al. 1999) and the microglial population self-renews regularly throughout the lifespan (Askew et al. 2017; Manjally and Tay 2022; Réu et al. 2017; Ajami et al. 2007). The mechanisms of microglial renewal in the adult brain are debated (Huang et al. 2018; Elmore et al. 2014; Bruttger et al. 2015). Elmore et al. (2014) demonstrated rapid repopulation of microglia in the brains of male 18-mo WT C57BL/6 mice following depletion of the microglial population by administration of 290mg/kg of PLX3397 for 28 days. Only 3 days following cessation of treatments, IBA1 positive microglia began to emerge and by 14 days microglia with ramified morphologies were present throughout the brain in equal amounts to that of age-matched untreated mice (Elmore et al. 2014). The newly formed microglia at 3 days expressed Ki67, indicating proliferation, and were positive for the neuroectodermal development marker nestin. Notably, subsequent analysis revealed that microglial repopulation was not due to infiltration of circulating monocytes, with fate mapping using BrdU labelling of proliferative non-microglial cells revealing that these cells went on to become microglia. These cells were also positive for nestin, suggesting that they may represent a population of microglial progenitor cells (Elmore et al. 2014).

Nonetheless, evidence suggests that this method of microglial renewal occurs mainly following mass depletion of the microglial population, with previous studies in mice demonstrating that microglia are generally long lived, with half the microglial population lasting the entire lifespan of an animal (Füger et al. 2017). In support of this, a study using 3-mo and 22-mo male C57BL/6 mice demonstrated that depleting microglia by PLX3397 administration for 7 days and allowing rapid repopulation from these hypothesised progenitor cells led to a microglial population in 22-mo ‘repopulated’ mice which resembled that seen in 3-mo controls (Elmore et al. 2018). This suggests a different ageing profile compared to microglia which have undergone slow turnover throughout the lifespan (Réu et al. 2017). Similarly, a study in human post-mortem tissue from young (20- to 35-years-old) and old (58- to 79-years-old) adults showed no changes in the number of microglia positive for the proliferative marker Ki67 between groups. This supports the hypothesis of slow turnover across the lifespan, rather than a rapid regeneration of microglia from a progenitor population (Askew et al. 2017).

Across the lifespan, the microglial population may be depleted by insults such as infections, injuries or neurodegenerative pathology. Indeed, the presence of amyloid pathology in a triple-transgenic CD11b-CreERT2;R26-tdTomato;APP/PS1 mouse model of AD leads to steady loss of microglia and compensatory proliferation (Füger et al. 2017). Interestingly, utilising a cranial window for visualisation of fluorescently labelled microglia, newly appearing cells were observed to be preceded by increased cell body volume of the presumed origin cell, suggesting proliferation from existing microglia, rather than a progenitor population (Füger et al. 2017). Overall, the literature as a whole supports that microglial renewal occurs across the lifespan through proliferation from mature cells, with the potential for regeneration from apparent progenitor cells following significant depletions in the microglial population (Hughes and Bergles 2014; Elmore et al. 2014, 2018). Whether this progenitor population exists in human brains, however, has not yet been fully established, and key questions remain around if the regenerative capacity of such cells is altered with age in mice.

### Altered Intercellular Signalling

Changes in intercellular communication are a hallmark of ageing, with alterations in cell signalling pathways potentially leading to an increased pro-inflammatory environment and loss of homeostatic processes (López-Otín et al. 2023). During ageing, there is dysregulation in several mechanisms of intercellular communication, including changes in pro-inflammatory signalling pathways. For microglia in particular, intrinsic changes in pathways such as the NF-κB/NLRP3, Fyn kinase and MAPK pathways lead to altered release of key signalling molecules, including cytokines. The NLRP3 inflammasome is expressed by both microglia (Cho et al. 2014) and astrocytes (Lu et al. 2014) in the CNS, but is primarily associated with microglia (Chiarini et al. 2023). Under physiological conditions, NLRP3 is expressed at low levels. However, during the inflammatory response, microglia have increased activation of the NLRP3 inflammasome complex (Fig. 3a) (Song et al. 2017). This leads to the release of IL-1β, in turn activating neighbouring cells, including other microglia, which further perpetuate the inflammatory response (Kamo et al. 2013). With age, the NLRP3 inflammasome becomes overactive, indicating a potential contribution to the chronic pro-inflammatory state seen in microglia with advancing age (Liang et al. 2024). In line with this, in 18-mo old male BALB/c mice, multiple NLRP3 pathway proteins, including ASC, caspase-1, IL-18 and NLRC4, were increased in the hippocampus and cortex compared to 3-mo mice (Mejias et al. 2018). Additionally, there was an increase in pyroptosis in these regions, indicating a role for increased NLRP3 signalling in pyroptotic cell death. Of note, microglial activation measured by assessment of microglial morphology in the dentate gyrus has been shown to decrease in 23-mo NLRP3^−^/^−^ mice compared to age-matched WT controls (Youm et al. 2013). Additionally, this measure decreased relative to 2-mo NLRP3^−^/^−^ mice, suggesting that NLRP3 activation may play a larger role in microglial dynamics with advancing age (Youm et al. 2013).

NF-κB signalling is involved in activation of the NLRP3 inflammasome by causing the transcription of pro-IL-1β, pro-caspase-1 and NLRP3 (Liu et al. 2017) (Fig. 3a). Age-related increases in NF-κB signalling and nuclear translocation lead to an increase in both NLRP3 inflammasome formation and secretion of pro-inflammatory cytokines (Liu et al. 2017). Figure 3a.4 shows the mechanisms by which NF-κB is translocated to the nucleus. IκB is normally bound to NF-κB in the cytoplasm and prevents NF-κB activation. IκB phosphorylation and degradation by the proteasome exposes NF-κB localisation subunits, including P50 and P65, leading to translocation of NF-κB to the nucleus, where it then triggers various gene expression (Liu et al. 2017). Alterations in these NF-κB subunits, specifically ablation of the p50 subunit in C57BL/6J mice (p50^−^/^−^), has been shown to increase the inflammatory response of microglia to LPS administration (Taetzsch et al. 2019). This response was exacerbated in p50^−^/^−^ mice at 16–18-mo compared to both p50^+^/^+^ age-matched controls and young p50^−^/^−^ mice (3-weeks-old), suggesting that NF-κB disruption may be particularly detrimental in the context of ageing (Taetzsch et al. 2019).

Fyn kinase and MAPK pathways are upstream regulators of both NLRP3 and NF-κB, with increased Fyn and p38 MAPK signalling promoting pro-inflammatory signals within microglia (Bachstetter et al. 2011; Panicker et al. 2015). Previous literature indicates that activation of both pathways may be impacted by ageing, although in different ways. One study investigated Fyn kinase signalling in 12-mo C57BL/6J mice following traumatic stress induced by dorsomyotomy and exploratory laparotomy (Zhao et al. 2012). This study demonstrated reductions in Fyn signalling in both pre- and post-synaptic density protein fractions in the frontal cortex of aged mice compared to 2-mo mice three days following surgical procedure (Zhao et al. 2012). This suggests that Fyn signalling following traumatic stress is further altered in an aged environment. Conversely, in male C57BL/6J mice at 20-mo of age, phosphorylated-p38 MAPK protein levels in the brain were increased compared to 2-mo mice (Li et al. 2011). While neither Fyn nor MAPK pathways have yet been studied specifically in microglia during ageing, given these are key immune pathways in microglia, it is reasonable to hypothesise that dysregulation of these pathways may be responsible for the upregulation in pro-inflammatory signalling seen in microglia with increased age. Understanding these pathways in microglia is critical for identifying microglial signalling pathways which contribute to dysfunction with ageing.

### Chronic Inflammation

Evidence for chronic inflammation in the aged brain has previously been extensively reviewed (Andronie-Cioara et al. 2023; Jin et al. 2022; Moyse et al. 2022). Given their critical role in regulating the inflammatory response, microglia are likely a major driver of this response. Notably, microglia in aged mice sustain the inflammatory response for longer following brain injury than their young counterparts. For example, following a controlled cortical impact (CCI), microglia in the hippocampus of male 24-mo C57BL/6 mice showed increased relative expression of Cd11b and IBA1 (markers of the microglial immune response) following injury, which remained heightened at 14 days post-injury (Sandhir et al. 2008). Comparatively, these markers in adult mice at 5- to 6-mo were expressed at lower levels at all timepoints and returned to baseline expression levels by 14 days post-injury (Sandhir et al. 2008). Additionally, a separate study has shown that aged C57BL/6 male mice display a propensity to acquire a pro-inflammatory phenotype at 24-months of age following CCI. These mice demonstrated a decreased ability to mount an anti-inflammatory response compared to 3-mo mice (Kumar et al. 2013). Similar results have also been seen in stroke models, where, following intracerebral haemorrhage induced by collagenase injection to the striatum, male Sprague–Dawley rats at 22-mo showed a slower microglial response and prolonged microglial immune activation around the hematoma compared to 3- to 4-mo rats following injury (Wasserman et al. 2008). This study additionally demonstrated morphological differences in microglia of naïve animals with age, with microglia from 24-mo rats demonstrating shorter and thicker processes, characteristic of bushy morphology, compared to the ramified morphology in 3- to 4-mo rats.

The population of microglia is also altered with age; however, there appear to be clear sex differences in these changes. For example, in the hippocampus of male C57BL/6 mice, microglial number was stable throughout the lifespan, with no changes observed from young (4- to 5-mo) to middle-aged (13- to 14-mo) to aged (27- to 28-mo) mice in the dentate gyrus, CA1 or Hilus (Long et al. 1998). Additionally, no changes in microglial density in these regions were observed, nor were there differences in the number of astrocytes present, indicating that glial numbers are stable with age in males, at least within hippocampal regions. Conversely, in a separate study by the same authors, in the CA1 region of female C57BL/6 mice, microglial numbers increased steadily across young (3- to 4-mo), middle-aged (13- to 14-mo) and aged (20- to 24-mo) cohorts (Mouton et al. 2002). Importantly, at each timepoint, the microglial counts in female mice were significantly higher than CA1 counts in the equivalent male cohort from the previous study. These differences are also seen in human post-mortem brains, where the number of microglia in the female neocortex (age range: 18–93 years) has been shown to increase with age, despite no such changes observed in these regions in males (age range: 19–87 years) (Pelvig et al. 2008). Taken together, these results indicate an effect of biological sex on microglial dynamics with age and thus highlight the importance of including sex-balanced cohorts in ageing research.

Beyond population dynamics, the phenotype of microglia is also altered with increased age. In line with this, the number of microglia initiating an immune response, as measured by MHCII positivity, increased with age in both mouse and non-human primate tissue. In male C57BL/6 mice, the number of Cd11b/MHCII positive microglia at 22- to 24-mo was increased compared to in 3- to 5-mo mice (Garner et al. 2018). Similarly, when investigated in macaque brains, microglial MHCII expression increased at 10- to 18-years-old compared to at 2- to 5-years-old (Sheffield and Berman 1998), indicating that these changes are not only seen in rodent brains but also in the brains of higher order mammals. In humans, microglial immune activation with age has been studied by utilising the positron emission tomography (PET) ligand (R)-[^11^c]PK111955, which specifically binds to inflammatory microglia expressing the 18 kDa translocator protein (TSPO) (Shah et al. 1994). One study using this ligand in a cohort of 33 subjects (22M:11F) aged 19- to 79-years-old found an association between increasing age and enhanced ligand binding throughout the brain. This data suggests that activation of microglial inflammatory pathways is associated with increasing age in healthy living subjects (Schuitemaker et al. 2012). Additionally, in the HuMi_Aged dataset, interferon signalling pathways were enriched and upregulated compared to microglia from a middle-aged dataset (Olah et al. 2018; Zhang et al. 2016). Taken together, these data support a pattern of increased inflammatory microglia with age, regardless of species and sex. This altered microglial phenotype may contribute to the ongoing chronic inflammation seen in age-related NDs.

Age-related intrinsic changes within microglia are thought to drive the chronic inflammatory environment observed in the aged brain. Nuclear and mtDNA damage is thought to play a key role in this through the DNA-driven immune response (Pérez-Treviño et al. 2020). In line with this, in 20-mo C57BL/6J mice, STING activity was shown to be upregulated in microglia, suggesting heightened cytosolic DNA sensing in these cells (Gulen et al. 2023). Indeed, the increased stimulation of the cGAS-STING pathway, described in the mitochondrial dysfunction section above, is thought to drive the shift towards a pro-inflammatory phenotype with age, contributing to ongoing inflammation in microglia (Gulen et al. 2023). These studies support the idea that microglia are a driver of the chronic inflammation observed with advancing age, which potentially underlies and likely exacerbates ND pathology.

### Dysbiosis

Dysbiosis as a hallmark of ageing refers to the imbalance of bacteria in the gut (López-Otín et al. 2023). A 2022 review identified two groups of taxa associated with ageing (Ghosh et al. 2022). The first group consisted of mainly pro-inflammatory taxa and was associated with unhealthy ageing, defined by shortened lifespans, whereas the second group consisted of beneficial taxa and was associated with healthy ageing. Notably, levels of beneficial taxa were decreased in unhealthy ageing (Ghosh et al. 2022). This suggests that longevity and healthy ageing may be associated with higher levels of beneficial taxa in the gut, while pro-inflammatory taxa may lead to an accelerated ageing process. Links between changes in the composition of the gut microbiome and CNS conditions are becoming increasingly apparent (Abdel-Haq et al. 2019), and the gut–brain axis is an important pathway of regulation of glial function (Loh et al. 2024).

Microglia appear to be the most vulnerable CNS cell type to changes in the gut microbiome (Huang et al. 2023). For example, in 2-mo Kun Ming mice, microglial gene expression in response to altered gut microbiome was altered more than in any other cell type (Huang et al. 2023). Specifically, differential expression of inflammasome genes, genes associated with mitochondrial dysfunction and oxidative phosphorylation genes was noted in both the prefrontal cortex and hippocampus (Huang et al. 2023). Given this, it is reasonable to hypothesis that microglial function and maturation may be influenced by age-related changes in the gut microbiota. In line with this, indirect modulation of microglia via interactions of pro-inflammatory bacteria with peripheral immune cells has been demonstrated in aged mice. Peripheral administration of LPS in 20-mo male BALB/c mice induced a hyperactive microglial response, as measured by increased mRNA expression of IL-1β, IL-10 and TLR2 in isolated microglia (Henry et al. 2009). Given that LPS is often released as a signalling byproduct by microbes in the gut (Mohr et al. 2022) and that increased gut permeability in older age allows LPS to cross into blood circulation (Thevaranjan et al. 2017), this study may implicate age-related pro-inflammatory changes in the gut as a potential moderator of microglial response. To date, however, there are limited studies on the impact of specific age-related microbial alterations in the gut-brain axis on microglia. Nevertheless, the evidence that microglia can be disproportionately affected by changes in the gut suggests that this could be a potential area for intervention to reduce aberrant age-related changes in microglia.

## Beyond Immune Activation: Unresolved Questions and Future Directions for Microglial Ageing

**Fig. 4:**
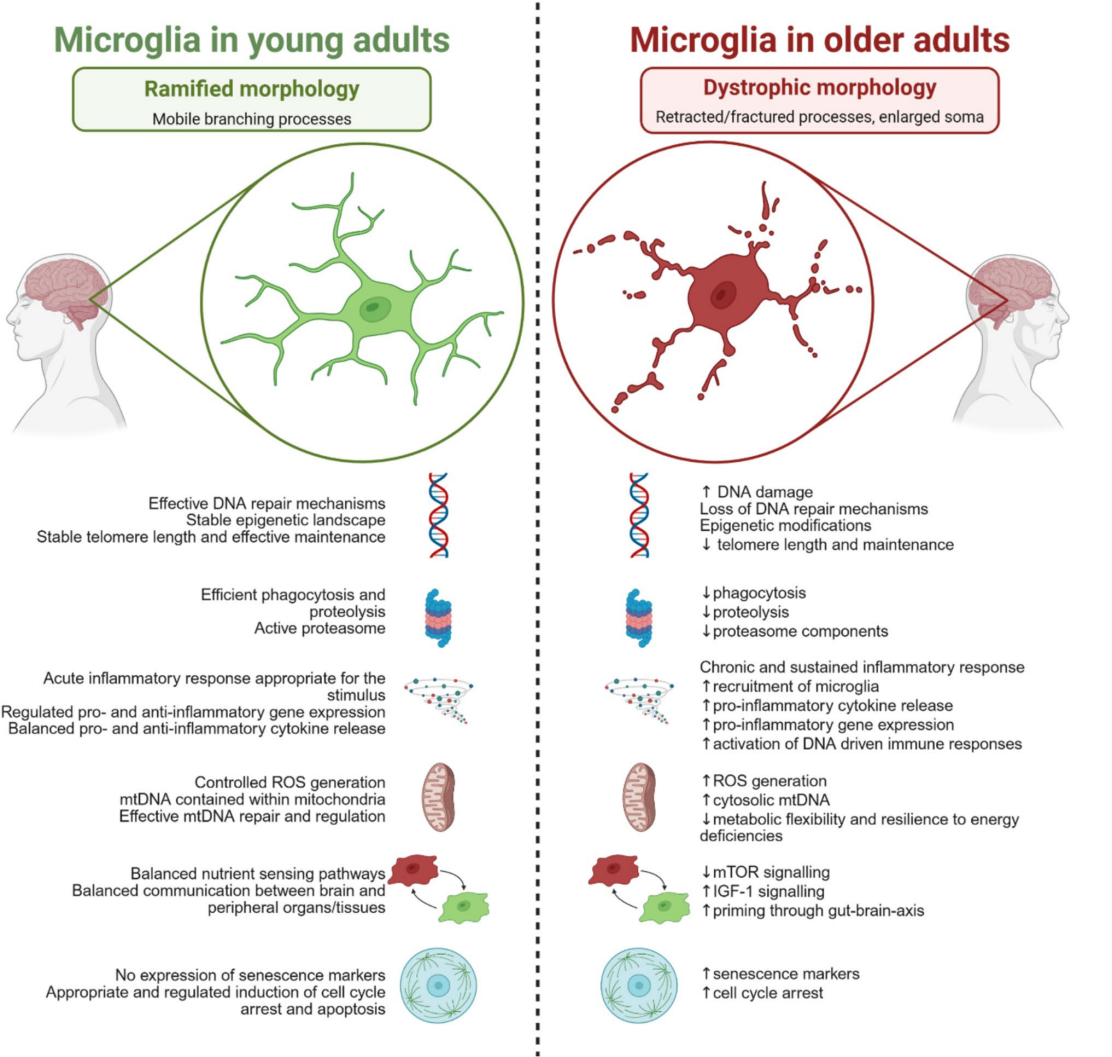
Features of adult (left) and aged (right) microglia. Left: Normal young adult microglia are highly motile with a ramified morphology and can perform homeostatic and immune functions without inducing hostile environmental conditions. Right: Aged microglia display enlarged dystrophic morphology and present with many of the classic hallmarks of ageing

Overall, the dystrophic morphology of aged microglia is accompanied by the acquisition of a senescent state with increased pro-inflammatory signalling. The aged phenotype of microglia as related to the twelve hallmarks of ageing is outlined in Fig. 4. Microglia display a decreased capacity for homeostatic functions, including phagocytosis, and an increased production and release of pro-inflammatory factors, resulting in increased pro-inflammatory signalling. In general, this decreased capacity of microglia to maintain homeostatic immune functions may contribute to the vulnerability of the brain to age-related diseases, including the development of NDs (Koellhoffer et al. 2017). However, these changes may not be the sole cause of NDs, and indeed, age-related changes in neurons also appear to be major drivers of disease pathophysiology (Höhn et al. 2020).

While the studies discussed above highlight significant features of aged microglia, this is still a relatively understudied area. Research on ageing in peripheral macrophages has garnered more attention, with the hallmarks of ageing in macrophages reviewed recently (Guimarães et al. 2021). However, microglia have not received equivalent focus, a notable gap which is likely due to the challenges of accurately replicating aged CNS cells in research models (Carr et al. 2024). This is also reflective of the fact that ageing more generally is often not considered in pre-clinical modelling studies of ND, a critical consideration to improve translational potential (Carr et al. 2024). Tables 2 and 3 summarise studies conducted to date which have investigated each hallmark of ageing in microglia in either experimental models or human tissue, respectively.

**Table 2:** Evidence of the hallmarks of ageing in microglia from laboratory models

Hallmark	Features seen in aged microglia				
	Model	Sex	Ages compared Age of observed change is underlined	Changes observed	Ref
Genomic instability	C57BL/6 mice	Not specified	24 mo compared to 2 mo	↑accumulation of DNA damage measured by γ-H2A.X staining ↑ percentage of microglia positive for pSTING and CD11b	(Arvanitaki et al. 2024)
	*Ercc1*^*Δ/−*^mice on C57BL6J/FVB background	Male and female	16wo compared to age/sex matched genetic controls	Hypertrophy of processes and ↑ cell body size ↑proliferation, ↑phagocytosis, ↑ROS production	(Raj et al. 2014)
	*Ercc1*^*Δ/−*^mice induced by tamoxifen administration at 6- to 8-wo on C57BL6J/FVB background	Male and female	2-, 6-, and 12-months following tamoxifen administration (approx. 4-, 6-, and 14-mo) compared to age/sex matched genetic controls	↓microglial number at 6- and 12-month timepoints	(Zhang et al. 2021)
Telomere attrition	Microglia isolated from cortex of neonatal rats	Not specified	Aged by replicative senescence over 32 days in culture	↓telomere length measured by flow-FISH Steady decline and then rapid increase in telomerase activity during culture	(Flanary and Streit 2004)
	Microglial isolated from Fisher-344 rats	Male	30-mo compared to 3-mo	↓telomere length measured by flow-FISH ↓telomerase activity	(Flanary et al. 2007)
	Terc knockout mice with AD pathology: G3Terc^−/−^APP23^+^ mice	Not specified	12-mo compared to age-matched genetic controls	↓Aβ pathology in frontal cortex Improved spatial memory No microglial immune activation near Aβ plaques	(Rolyan et al. 2011)
	Terc knockout mice with PD pathology: αSYN^tg/tg^G3Terc^−/−^ mice	Not specified	75-wo compared to age-matched genetic controls	↓microglial IL-1β and MHCII in the brainstem	(Scheffold et al. 2016)
Epigenetic changes	Microglia isolated from C57BL/6J mice following LPS exposure	Not specified	24- to 26-mo compared to 4- to 6-mo	↓ methylation of IL-1β promoter DNA leading to increased IL-1β production	(Matt et al. 2016)
	C57BL/6J mice	Male	16- to 18-mo compared to 2- to 3-mo	↑ iNOS, IL-6 and TNF ↑ H3K27me3 in the midbrain ↓ JMJD3	(Tang et al. 2014)
	C57BL/6J mice	Male and female	24-mo compared to 3- and 14-mo	↑ chromatin accessibility of phagocytosis, IFN signalling, ROS production and antigen presentation genes ↓ chromatin accessibility of chemokine suppression and iron transportation genes	(Li et al. 2023a)
Loss of proteostasis	Microglia isolated from C57BL/6N mice	Not specified	20- to 24-mo compared to 3- to 5-mo	↓ proteasomal subunits PSMB4, PSMA6, PSMC1	(Flowers et al. 2017)
	APP/PS1 mice lacking the LMP7 immunoproteasome component	Not specified	8-mo compared to age-matched genetic controls	↓ microglial immunoreactivity No change in Aβ pathology	(Wagner et al. 2017)
	Deletion of E3 ubiquitin ligase COP1 in C57BL/6N mice by tamoxifen administration	Male and female	22-mo compared to young WT mice	↑ microglial IFN signalling gene expression ↑APOE expression	(Ndoja et al. 2020)
	C57BL/6 mice	Male and female	Sex differences at 18-mo compared to 3-mo Both sexes differences at 22-mo compared to 3-mo	Female mice: ↓ microglial phagocytic action on Aβ42 Male mice: ↓ proteolytic gene expression Both sexes: ↓ expression of phagocytic receptor TREM2	(Thomas et al. 2022)
Disabled macroautophagy	Microglia isolated from mice with deletion of WD domain of Atg16L on a mixed 129/C57BL/6 background	Male and female	24-mo compared to age-matched genetic controls	↓ uptake of Aβ	(Heckmann et al. 2020)
	Bone marrow derived macrophages isolated from C56BL/6 mice**	Male	23-mo compared to 6- to 8-wo	↓expression of autophagic genes ATF7 and ATG-6 Impaired autophagic flux	(Stranks et al. 2015)
Deregulated nutrient sensing	Microglia isolated from C57BL/6N mice	Not specified	20- to 24-mo compared to 3- to 5-mo	↓ mTORC2-dependent AKT D473 phosphorylation ↑IL-6, IL-1β, TNFα at baseline and in response to TNFα	(Flowers et al. 2017)
	Wistar rats treated with insulin for 5 days	Male	22-mo compared to 3-mo	↓IBA1 and CD68 positive microglia in the hippocampus and no induction of COX-2/IL-1β immune response	(Haas et al. 2020)
	Microglia isolated from C57BL/6J mice	Female	23-mo	mTOR dysregulation	(Keane et al. 2021)
	APP_SWE_/PS1_ΔE9_ mice	Female	24-mo compared to age-matched genetic controls	↑IGF-1 and TNF mRNA in hippocampus ↑IGF-1 mRNA-expressing microglia in subgranular zone of dentate gyrus	(Myhre et al. 2019)
Mitochondrial dysfunction	WT C57BL/6 mice and C57BL/6 mice overexpressing TFAM	Male	24-mo WT compared to 2-mo WT 24-mo TFAM compared to 2-mo WT	24-mo WT: ↑lipid peroxidation 24-mo TFAM: ↓ lipid peroxidation	(Hayashi et al. 2008)
	Microglia isolated from C57BL/6 mice	Not specified	20- to 24-mo compared to 2- to 4-mo	↑PGE2-EP2 signalling leading to enhanced glycogen storage and ↓mitochondrial respiration Inhibition of EP2 signalling restored metabolic function *changes also relevant to dysregulated nutrient sensing due to altered glucose handling via PGE2-EP2 signalling	(Minhas et al. 2021)
	Microglia isolated from C57BL/6 mice	Not specified	20-mo compared to 8- to 12-wo	↑cytosolic mtDNA	(Gulen et al. 2023)
Cellular senescence	C57BL/6 mice	Male	18-mo compared to 3-mo	↑ expression of p16^INK4A^ but no change in p21^Cip1/Waf1^ Accumulated lipofuscin ↑ DNA damage measured by γ-H2A.X staining	(Ritzel et al. 2019)
	C57BL/6 mice	Male and Female	24-mo	Accumulated lipofuscin	(Stillman et al. 2023)
	5XFAD mice and C57BL/6J WT mice	Male and female	8-mo compared to 4-mo	↑presence of SA-β-Gal^+^ microglia proximal to Aβ plaques	(Shin et al. 2024)
	BV2 microglia-like cells	N/A	Cells aged by repetitive (6x) LPS administration	↑SA-β-Gal and ↑SAHF formation	(Yu et al. 2012)
	Transgenic p7.2*fms-*EGFP mice on a C57BL6/6XCBA background	Male and female	18-mo compared to 2-mo	↑pro- ((IL-6, TNFα, IL-1β) and anti- (IL-10) inflammatory cytokines	(Sierra et al. 2007)
	Microglia isolated from spinal cords of C57BL/6J mice	Male	22-mo compared to 3-mo	↑TNFα, IL-1β release ↑ ROS-induced oxidative stress ↑microglia phagocytic ability	(Ritzel et al. 2015)
Stem cell exhaustion	Cd11b-CreERT2 transgenic mice on a C57BL/6J x C3Hej background	Male and female	15-mo compared to 4-mo	Microglial cells are long lived and stable across life	(Füger et al. 2017)
	C57BL/6 mice	Not specified	18- to 20-mo compared to 4- to 6-mo	No changes in microglial number in hippocampus or cortex but ↑ in the thalamus	(Askew et al. 2017)
Altered intercellular communication	BALB/c mice	Male	18-mo compared to 3-mo	↑ NLRP3 pathway proteins in hippocampus and cortex ↑pyroptosis in hippocampus and cortex	(Mejias et al. 2018)
	NLRP3^−^/^−^ mice	Male and female	23-mo compared to 2-mo and to aged-matched genetic controls	Loss of NLRP3 caused ↓ in microglial immune activation in dentate gyrus ↓in IL-1β and TNF mRNA expression	(Youm et al. 2013)
	C57BL/6J mice deficient in the p50 NF-κB subunit	Not specified	16- to 18-mo compared to 3-wo and age-matched genetic controls	↑inflammatory response of microglia to LPS administration	(Taetzsch et al. 2019)
	C57BL/6J mice	Not specified	12-mo compared to 2-mo	↓Fyn in pre- and post-synaptic protein fractions from the frontal cortex following traumatic stress	(Zhao et al. 2012)
	C57BL/6J mice	Male	20-mo compared to 2-mo	↑phospho-p38 MAPK in brain tissue	(Li et al. 2011)
Chronic inflammation	C57BL/6 mice following a moderate controlled cortical impact (CCI)	Male	21- to 24-mo compared to 5- to 6-mo	↑ inflammatory response in the hippocampus following injury ↑ time to resolve response	(Sandhir et al. 2008)
	C57BL/6 mice following CCI	Male	24-mo compared to 3-mo	↑pro- and ↓ anti-inflammatory markers of microglia	(Kumar et al. 2013)
	Sprague–Dawley rats following collagenase-induced intracerebral haemorrhage	Male	22-mo compared to 3- to 4-mo	Slower microglial response to injury Prolonged immune activation around hematoma	(Wasserman et al. 2008)
	C57BL/6J mice	Male	27- to 28-mo compared to 13- to 14-mo and 4- to 5-mo	No change in microglial number in hippocampal regions across ageing	(Long et al. 1998)
	C57BL/6J mice	Female	20- to 24-mo compared to 13- to 14-mo and 3- to 4-mo	Increase in microglial numbers with age in CA1 region Increased numbers at each age group compared to equivalent cohort in males (study above (253))	(Mouton et al. 2002)
	C57BL/6 mice	Male	22- to 24-mo compared to 3- to 5-mo	↑ MHCII positive microglia	(Garner et al. 2018)
	*Macaca nemestrina*	Females	10- to 18-yo compared to 2- to 5-yo	↑ MHCII positive microglia	(Sheffield and Berman 1998)
	C57BL/6J mice	Not specified	20-mo compared to 3-mo	↑ STING activity	(Gulen et al. 2023)
Dysbiosis	BALB/c mice	Male	18- to 20-mo compared to 3- to 4-mo	Peripheral administration of LPS leads to hyperactive immune response ↑mRNA expression of IL-1β, IL-10 and TLR2	(Henry et al. 2009)

^**^studies conducted in macrophages NOT microglia

**Table 3:** Evidence of the hallmarks of ageing in microglia from human samples

Hallmark	Features seen in aged microglia				
	Model	Sex	Ages compared Age of observed change is underlined	Changes observed	Ref
Genomic instability	Human post-mortem tissue from the: temporal cortex, hippocampus, entorhinal cortex, substantia nigra, superior colliculi, locus coeruleus and inferior colliculi	6M:8F	65–96 years old (80.42 ± 9.07)	Dystrophic microglia associated with ↑ferritin but not γ-H2A.X staining	(Neumann et al. 2023)
	Microglia isolated from human dorsolateral pre-frontal cortex (HuMi_Aged dataset)	10F	94.07 years ± 0.95 compared to published middle age microglial transcriptomic dataset (53 years ± 5.29)	Identified 1060 upregulated, and 1174 downregulated genes with ageing ↑chromosomal maintenance pathways	(Olah et al. 2018)
Telomere attrition	Microglia isolated from human superior frontal gyrus/frontal pole region	AD = 3M:1F ND = M	4 AD cases (86-years-old ± 2.54) One non-dementia case (86-years-old)	↓telomere length measured by flow-FISH No change in telomerase activity	(Flanary et al. 2007)
Epigenetic alterations	HuMi_Aged dataset	10F	94.07 years ± 0.95	Analysis of REACTOME pathways revealed enrichment of DNA methylation pathways Upregulation of DNA methylation and histone deacetylase pathways compared to microglia from middle age (53 years old) dataset	(Olah et al. 2018)
Loss of proteostasis	HuMi_Aged dataset	10F	94.07 years ± 0.95	ER-phagosome pathway enriched in HuMi dataset and upregulated compared to middle-aged microglia (53 years old)	(Olah et al. 2018)
Disabled macroautophagy	No studies investigating human microglia during ageing				
Deregulated nutrient sensing	No studies investigating human microglia during ageing				
Mitochondrial dysfunction	No studies investigating human microglia during ageing				
Cellular senescence	HuMi_Aged dataset	10F	94.07 years ± 0.95	Enrichment of telomere induced senescence pathway and SASP pathways	(Olah et al. 2018)
Stem cell exhaustion	Human post-mortem tissue	Not specified	58- to 79-yo compared to 20- to 35-yo	No changes in number of proliferating (Ki67^+^) microglia	(Askew et al. 2017)
Altered intercellular communication	No studies investigating human microglia during ageing				
Chronic inflammation	Human post-mortem neocortex	Males and females	Age range females 18–93 years Age range males 19–87 years	Number of microglia increased with age in females but not males	(Pelvig et al. 2008)
	In vivo human brain (PET imaging)	Males and females (22M:11F)	19 to 79-years-old	↑ (R)-[^11^c]PK111955 ligand binding associated with ↑ age	(Schuitemaker et al. 2012)
	Microglia isolated from human right parietal cortex	N = 39, sex not specified	34 to 102-years-old	Top 100 genes most affected by ageing were enriched for immune response pathways	(Galatro et al. 2017)
	HuMi_Aged dataset	10F	94.07 years ± 0.95	Interferon signalling pathways enriched in HuMi dataset and upregulated compared to middle-aged microglia (53 years old)	(Olah et al. 2018)
Dysbiosis	No studies investigating human microglia during ageing				

Much of the literature discussed in the current review is based on investigations in small animal models, with limited research conducted in aged microglia in human samples to date. Notably, for several hallmarks (i.e. disabled macroautophagy, dysregulated nutrient sensing, mitochondrial dysfunction, altered intercellular communication and dysbiosis), we could not identify any human studies investigating microglia-specific changes with age. Further, while one transcriptional study by Olah et al. (2018) identified changes related to epigenetic alterations, loss of proteostasis and cellular senescence, beyond this singular study, there was also a lack of research examining these hallmarks in human microglia specifically. The limited literature on human microglia with age is likely due to the difficulties associated with obtaining human tissue (Rush et al. 2024). Unlike in laboratory models, where brain tissue can be collected at controlled timepoints, studying human microglia relies on post-mortem samples, which can be difficult to obtain, particularly for healthy older adults. Additionally, variables which may influence data, including post-mortem interval and pre-existing pathology, particularly in aged individuals, further complicates this analysis. To combat these challenges, future research should make use of in vivo imaging techniques, including positron emission tomography (PET, which has been utilised to assess human brain ageing and neuroinflammation ((R)-[^11^c]PK111955 (Schuitemaker et al. 2012), [^18^F]FDG (Xue et al. 2024; Doering et al. 2023), ^18^F-AV-1451 (Schöll et al. 2016)). Although such tracers differ in their specificity to microglia (reviewed: (Beaino et al. 2021)), they nevertheless present opportunities to elucidate age-related brain changes without reliance on post-mortem tissues.

In addition, there is an underappreciation of sex-specific differences in microglial ageing and immune activation. Previous literature has highlighted sex differences in microglia with age and in neuroinflammation more generally. For instance, in female Fischer 344 rats, microglial number in the dentate gyrus increases with age at 18-mo compared to in rats at 3-mo, but no difference is observed in male rats (Perkins et al. 2018). Similarly, in human post-mortem tissue, no changes in the number of microglia in the neocortex has been observed with age in males (19 to 87 years), but increased numbers have been seen in females (18 to 93 years) (Pelvig et al. 2008). These studies suggest sex-specific differences in microglial dynamics, which have been further explored and reviewed (Cyr and de Rivero Vaccari 2023; Lynch 2022) and have been suggested as a driving mechanism for broader sex differences seen in ND susceptibility (Lynch 2022). This is of concern, as only 20% of pre-clinical neuroscience studies in rodents use both sexes and 25% do not report the sex of subjects (Beery 2018). Future research should, therefore, endeavour to consistently include and report data for both female and male animals, in accordance with the Animal Research: Reporting of In Vivo Experiments (ARRIVE) guidelines (Percie du Sert et al. 2020) in order to better understand the influence of sex on age-related changes in microglia.

Beyond a general lack of knowledge regarding microglial expression of key hallmarks of ageing, the current review highlights the inconsistencies in classifying microglia, a well-documented issue in the field (Paolicelli et al. 2022; Reddaway et al. 2023; Green et al. 2022). Previous reviews have discouraged the use of outdated terms, such as resting and activated (Paolicelli et al. 2022) and outlined the lack of consensus regarding morphological classifications of microglia (Reddaway et al. 2023). While extensive discussion of this issue is beyond the scope of the current review, in order to work towards a cohesive understanding of aged microglia and move the field forward, this issue must be addressed. When classing populations of microglia, future studies should refer to functional classifications, rather than relying solely on morphological profile. Further, as outlined by (Paolicelli et al. 2022), it is critical that the wider scientific community collaborate to establish a unified approach to microglial analysis and classification potentially through the use of complex analyses, such as single-cell proteomics and transcriptomics.

## Conclusions

As the major immune cells of the brain, microglia have a critical role in maintaining brain health and responding to immune challenges. Ageing has a profound impact on microglial function and intrinsic changes within these cells are suggested to contribute to vulnerability of the brain to age-related disorders, including NDs. Currently, the literature defines aged microglia as pro-inflammatory and dystrophic, with a similar phenotype to that seen in disease. However, studies on microglial morphology may benefit from greater consistency of terminology and clear definition of microglial phenotypes. In addition, studies have largely focused on pro-inflammatory processes linked to microglia in NDs or following injury. However, understanding the changes which occur in microglia with physiological ageing alone is critical in understanding the contribution of the ageing process to disease susceptibility and progression.

Here, a consideration of alterations in the 12 hallmarks of ageing (López-Otín et al. 2023) within microglia highlights clear gaps in our knowledge of how these processes change in microglia with increasing age. Many of these processes have been investigated in the context of disease (e.g. NDs), but studies have only rarely investigated the impact of ageing alone on these hallmarks. For example, both chronic inflammation and changes in proteostasis within microglia have been extensively studied in the context of ND. Conversely, other hallmarks not as clearly associated with ND pathophysiology, such as gut dysbiosis and stem cell exhaustion, have been investigated in aged microglia in only a handful of studies to date. Thus, the full phenotype of aged microglia remains poorly characterised and needs to be better understood to improve our ability to capture these changes in research models. As such, a comprehensive characterisation of microglia at different points across the lifespan is needed to understand how these cells contribute to vulnerability to disease, which may lead to the identification of novel therapeutic targets to reduce detrimental effects related to microglial ageing.

## Data Availability

No datasets were generated or analysed during the current study.
